# A National Study of Somatotypes in Mexican Athletes Across 43 Sports

**DOI:** 10.3390/jfmk10030329

**Published:** 2025-08-27

**Authors:** Ximena Martínez-Mireles, José Omar Lagunes-Carrasco, Vianney Curiel-Cervantes, Ximena Ortega-Salinas, Mauro E. Valencia, Ricardo López-García, Silvia García, Cristina Bouzas, Rogelio Salas-García, Erik Ramírez, Josep A. Tur

**Affiliations:** 1 Facultad de Salud Pública y Nutrición, Universidad Autónoma de Nuevo León UANL, Av. Universidad S/N Ciudad Universitaria, San Nicolás de los Garza 66451, Mexico; ximenamtznutricion@gmail.com (X.M.-M.); erik.ramirezlp@uanl.edu.mx (E.R.); 2 Facultad de Organización Deportiva, Universidad Autónoma de Nuevo León UANL, Av. Universidad S/N Ciudad Universitaria, San Nicolás de los Garza 66451, Mexico; 3 Secretaría de Ciencia, Humanidades, Tecnología e Innovación SECIHTI, Universidad Nacional Autónoma de México, UNAM, Campus Juriquilla, Blvd. Juriquilla 3001, Juriquilla, Querétaro 76230, Mexico; viana.ccjv@gmail.com; 4 Instituto del Deporte y la Recreación del Estado de Querétaro INDEREQ, Blvd. Bernardo Quintana, S/N, Col. Villas del Parque, Querétaro 76090, Mexico; 5 Centro de Investigación en Alimentación y Desarrollo A.C. CIAD, Departamento de Nutrición, Carretera Gustavo Enrique Astiazarán Rosas 46, La Victoria, Hermosillo 83304, Mexico; 6 Research Group on Community Nutrition & Oxidative Stress, University of Balearic Islands-IUNICS, 07122 Palma de Mallorca, Spaincristina.bouzas@uib.es (C.B.); 7 Health Research Institute of the Balearic Islands (IdISBa), 07120 Palma de Mallorca, Spain; 8 CIBER Fisiopatología de la Obesidad y Nutrición (CIBEROBN), Instituto de Salud Carlos III (ISCIII), 28029 Madrid, Spain

**Keywords:** anthropometric profile, physical status, somatochart, body composition, Mexican athletes

## Abstract

**Background**: In Mexico, research on somatotypes in athletes has primarily focused on team sports, taekwondo, climbing, and triathlon. However, the available evidence remains limited. Therefore, the purpose of this study was to determine the somatotype of Mexican athletes by sex, and to compare somatotype and body composition across sport macro-categories in 43 disciplines. **Methods**: Anthropometric measurements were conducted according to the International Society for the Advancement of Kinanthropometry (ISAK) protocol. Athletes who participated in regional, national, or international competitions between 2008 and 2024 were included. **Results**: A total of 889 Mexican athletes (477 males and 412 females) across 43 disciplines were evaluated. Among male athletes, the predominant somatotype was endomorphic mesomorph (52.4%), followed by balanced mesomorph (17.6%) and ectomorphic mesomorph (13.6%). Among female athletes, the most reported somatotypes were endomorphic mesomorph (24.5%), mesomorphic endomorph (24.0%), and mesomorph-endomorph (21.4%). Athletes in endurance sports showed significant differences for both sexes compared to those in power and skill-based sports for both sexes (*p* < 0.05). Among males, team sports showed the highest values for body mass, height, and body fat percentage (%BF), while mesomorphy was greatest in track and field and ectomorphy in sprint events. Among females, track and field athletes presented the highest values for body mass, height, %BF, mesomorphy, and endomorphy, whereas endurance athletes exhibited the highest ectomorphy values. **Conclusions**: The findings suggest that, compared to international athletes, Mexican athletes exhibited a higher endomorphic component. It is recommended that somatotype assessments should be incorporated into regular monitoring protocols at national sports centers and considered in physical training programs to optimize performance and reduce the risk of injury.

## 1. Introduction

Physical status refers to the physical characteristics of the body, including size, shape, and body composition [1]. Genetic factors, diet, training, and the specific demands of each sport influence this configuration. Somatotype, in turn, is a classification system that describes the shape and composition of the human body, applicable to both athletes and non-athletes. It was initially proposed by Sheldon et al. [2] and later adapted by Heath and Carter as the anthropometric somatotype, which adds measurements of skinfolds, girths, and bone diameters [3,4]. This model considers three main components: endomorphy, mesomorphy, and ectomorphy. Endomorphy refers to a body type with a higher proportion of body fat and a softer body with curves; mesomorphy describes a muscular and well-developed body structure; and ectomorphy characterizes individuals with a thin, linear physique, low muscle mass, and fat, giving them a slender appearance with long limbs. Although most individuals exhibit a combination of these components, one typically predominates, influencing their physical capabilities and athletic performance [5,6,7,8].

Among male athletes, a predominant mesomorphic profile has been observed, characterized by a strong body structure and higher muscle mass. In contrast, female athletes tend to exhibit a more endomorphic profile, marked by a higher accumulation of adipose tissue [9,10]. The application of somatotype analysis contributes to optimizing athletic performance, assessing the balance between fat and muscle mass, determining nutritional status, identifying physical profiles prone to injury, and adapting training to the specific demands of each sport [6,11,12,13,14,15,16,17,18]. Moreover, determining somatotypes across sports macro-categories enhances the identification of morphological patterns and the design of targeted training programs [19,20]. Genetically, mesomorphic and ectomorphic configurations are primarily inherited, while environmental factors such as diet and physical activity exert a higher influence on endomorphy [5,21,22]. From a functional perspective, mesomorphy is particularly favorable for the development of strength and power, whereas ectomorphy is associated with improved performance in endurance disciplines [17,23].

The classification of somatotypes among athletes from Latin America and Spain reveals a predominance of the endomorphic component, often in combination with mesomorphic traits, depending on the sport and playing position. For instance, Spanish futsal players [24] present endomorphy values of 3.8, which are lower compared to Brazilian players with 4.7 [25] and differ from Mexican soccer players with 4.3 [26]; this reflects a higher proportion of body fat in sports involving Latin ethnic populations. Additionally, other studies have reported differences in skinfold thickness across Latin American countries among female soccer players [27]. The field of sports science is still developing and consolidating in Latin America. The quality of research is affected by limited resources, language barriers (given that English dominates international research), and the need for greater interdisciplinary integration [28,29] which may contribute to the relatively lower number of studies addressing somatotype and body composition in athletes compared to other regions. Regarding body composition, evidence suggests that Mexican adults, particularly women, tend to have higher levels of adiposity compared to other ethnic groups [30]. Specifically, among Mexican American athletes, a higher accumulation of fat in the limbs has been observed compared to the trunk [31].

In Mexico, research on somatotype in athletes was primarily focused on team sports [26,32,33,34,35], as well as on individual disciplines such as taekwondo, climbing, and triathlon [36,37,38]. Although these studies have contributed valuable insights into the somatotype of Mexican athletes, the available data remain limited and are concentrated in a small number of sports disciplines. Nationally, the lack of updated and representative morphological references hinders the establishment of practical standards for coaches, selectors, nutritionists, and institutional sports programs. Understanding the somatotype of Mexican athletes would provide essential reference information on body composition, physical characteristics, and nutritional status, which are key elements for monitoring and optimizing athletic performance. Moreover, the absence of specific information on sex-based and discipline-specific differences limits the development of more effective strategies within the national sports context. Therefore, the aim of this study was (1) to determine the somatotype of Mexican athletes by sex and (2) to compare somatotype and body composition across sport macro-categories in 43 disciplines.

## 2. Materials and Methods

### 2.1. Study Design

This observational, cross-sectional, and descriptive-analytical study was conducted using data previously collected from Mexican athletes. The study followed the guidelines of the Strengthening the Reporting of Observational Studies in Epidemiology (STROBE) statement for cross-sectional studies [39].

### 2.2. Setting

Athletes from northern, central, and southern Mexico were included in the study. All participants competed at regional, national, or international levels between 2008 and 2024, and the recruitment and data collection were conducted. The data originated from athletes affiliated with public and private institutions, as well as from individual sources, all of whom provided informed consent for the use of their data for research purposes. The study protocol was approved by the Ethics and Research Committee of the Facultad de Salud Pública y Nutrición at the Universidad Autónoma de Nuevo León (UANL) (registration number 24-FaSPyN-SA-04; 11 June 2024). All athletes included in the study signed a written informed consent form, which explained the purpose of the research, the procedures involved, potential benefits and risks, and the confidentiality of their data. In the case of underage athletes, assent was obtained from the participants along with written consent from their parents or legal guardians [40].

To ensure confidentiality, each athlete was identified using a unique code to guarantee the anonymous handling of information. The protocol complied with the guidelines established by the NOM-012-SSA3-2012, “*Que establece los criterios para la ejecución de proyectos de investigación para la salud en seres humanos*” (translation: which sets the criteria for conducting health research projects involving human subjects) [41] in Mexico, as well as the Declaration of Helsinki [42], to ensure adherence to ethical principles in research.

### 2.3. Participants

Athletes from 43 sports across various regions of Mexico were included. For the analysis, sport macro-categories were considered within each discipline, including differences by event type, playing position, and competition format. Athletes participated in regional, national, and international competitions between 2008 and 2024, during which recruitment and data collection took place. Participants were selected through purposive sampling. From a total of 889 Mexican athletes, 477 male and 412 female athletes were analyzed, all of whom met the following inclusion criteria: (i) assessment conducted during pre-competition, general, specific, or pre-season phases; (ii) measurements taken between 2008 and 2024; (iii) age range between 14 and 35 years; and (iv) active participation in regional, national, or international competitions. To be classified as an athlete, individuals were required to be actively engaged in official competitions, to be part of a systematic training program, and to be formally registered with a recognized sports organization at the regional, national, or international level [43,44]. This definition ensured that all included participants met formal criteria for athletic representation and competition.

The athletes included in this study participated in one or more of the following competitions: Universiada Nacional, ranked among the top 10 in the Comisión Nacional de Cultura Física y Deporte (CONADE) Games; Central American and Caribbean Games, Junior Pan American Games, Pan American Games, World Championships, Junior World Championships, Youth Olympic Games, and the World Series of Team Roping. They were also members of or participants in events organized by the Liga Mexicana de Powerlifting, the Comisión Nacional Deportiva Estudiantil de Instituciones Privadas (CONADEIP), the Organización Nacional Estudiantil de Fútbol Americano (ONEFA), the Mexican National American Football Team, the Mexican National Rugby Team, and the Federación Mexicana de Rodeo.

Exclusion criteria were as follows: (i) inactive athletes, (ii) injured athletes, (iii) paralympic-level athletes, preschool- and school-aged individuals, and (iv) athletes participating in winter or extreme sports. Elimination criteria included (i) incomplete anthropometric measurements, (ii) body composition assessed during non-designated macrocycle phases, (iii) voluntary withdrawal from extreme somatotype characteristic participation, and (iv) atypical physiological conditions at the time of assessment (e.g., dehydration, illness).

### 2.4. Variables

The variables assessed included sex, body mass (kg), height (m), age (years), body fat percentage (%BF) (calculated using Equation (5) from Lean et al. [45] for females and Equation (2) for males; based on triceps skinfold, age, and body mass index), triceps skinfold (mm), subscapular skinfold (mm), suprailiac skinfold (mm), thigh skinfold (mm), flexed arm girth (cm), thigh girth (mm), humerus breadth (cm), femur breadth (cm), and the three somatotype components: endomorphy, mesomorphy, and ectomorphy [3].

### 2.5. Measurements

Body weight was measured using a SECA^®^ 813bt scale (Seca GmbH & Co. KG, Hamburg, Germany) with a precision of ±0.1 kg. Height was measured with a SECA^®^ 213 stadiometer (Seca GmbH & Co. KG, Hamburg, Germany) (±0.1 cm). Flexed arm and calf girths were measured using a Lufkin^®^ anthropometric tape (±0.1 mm; Cooper Industries, Houston, TX, USA). Triceps, subscapular, supraspinal, and thigh skinfolds were measured with a Slim Guide^®^ caliper (±1.0 mm; Creative Health Products, Ann Arbor, MI, USA). Humerus and femur bone breadths were assessed using a Lenart^®^ anthropometer (Lenart Instruments^®^; ±0.1 mm).

### 2.6. Bias

All measurements were taken in duplicate, and a third measurement was performed if the intra-evaluator technical error of measurement (TEM) threshold was exceeded [46]. Assessments were conducted following the measurement protocol of the International Society for the Advancement of Kinanthropometry (ISAK) [47] and were carried out by certified anthropometrists at Levels 1, 2, and 3. The intra-evaluator TEM was 2.60% for skinfolds, 1.18% for breadths, and 0.85% for girths. Anthropometric assessments were conducted prior to each athlete’s training session and after a minimum fasting period of 4 h.

### 2.7. Somatotype Calculation

Somatotype and its three components—endomorphy, mesomorphy, and ectomorphy—were calculated in a Microsoft^®^ Excel^®^ spreadsheet (Microsoft 365, Version 2508, Build 19127.20134, Microsoft Corporation, Redmond, WA, USA) using the equations proposed by Heath–Carter [3]; Appendix A.1. The classification into 13 somatotype categories [3] was performed using the NutriSolver^®^ software, version 1.0.0, Monterrey, N.L., Mexico [48].

#### Somatochart and Tables by Sports

The somatochart is a graphical tool used to visually represent and classify somatotypes based on established anthropometric measurements [3] within a two-dimensional plane [49]. The location of the somatotype on the somatochart was determined by two coordinates derived from the endomorphic, mesomorphic, and ectomorphic components. These were calculated using the X and Y axes, where the X-axis represented the difference between ectomorphy and endomorphy, and the Y-axis reflected mesomorphic predominance relative to the other two components. The equations can be found in Appendix A.2.

### 2.8. Macro-Categories by Sports

To facilitate a better understanding of the somatotype distribution among the analyzed athletes, sports disciplines were grouped into six functional macro-categories: team sports, combat sports, individual sports, track and field, endurance events, and sprint events. This categorization was structured based on functional and morphophysiological criteria, with the aim of identifying potential common somatotype patterns within groups sharing similar competitive characteristics [20]. Therefore, they were considered equivalent for somatotype characterization. The sports included in each macro-category are detailed and visually represented in the corresponding somatochart in the Supplementary Material (Figures S1 and S2).

Tables were created showing the mean and standard deviation for age, body mass (kg), height (cm), body mass index (BMI) (kg/m^2^), body fat percentage (%BF), and the three somatotype components (endomorphy, mesomorphy, and ectomorphy), stratified by sport discipline, position, and category. Data were organized separately for male and female athletes.

### 2.9. Statistics

Descriptive statistics (mean ± standard deviation) were used for continuous variables. Data normality was assessed using the D’Agostino–Pearson test and further verified through histogram distributions. To analyze the distribution of the three most frequent somatotypes by sex, the chi-square test (χ^2^) was applied along with the Marascuilo procedure to identify group differences.

As the assumption of normal distribution was not met, the Kruskal–Wallis test and Dunn’s post hoc test with Bonferroni correction were used to compare sport macro-categories. Results were reported as medians and interquartile ranges (Q1–Q3). Outliers were identified using Tukey’s method [50]. A value was classified as outside if it was lower than the first quartile minus 1.5 times the interquartile range (IQR) or higher than the third quartile plus 1.5 × IQR (inner fences). Far-out values were defined as those lower than the first quartile minus 3 × IQR or higher than the third quartile plus 3 × IQR (outer fences). Quantitative variables were treated as continuous for both descriptive and inferential statistical analyses. No recategorization or dichotomization of these variables was performed.

As no comparative hypotheses were formulated, the results are presented without adjustments and represent the main estimates required by the STROBE guidelines [39]. For all statistical tests, the significance level was set at *p* < 0.05. Statistical analyses were performed using NCSS 8 software (version 8.0.24, Kaysville, UT, USA) [51].

## 3. Results

### 3.1. Selected Athletes

**Figure 1:**
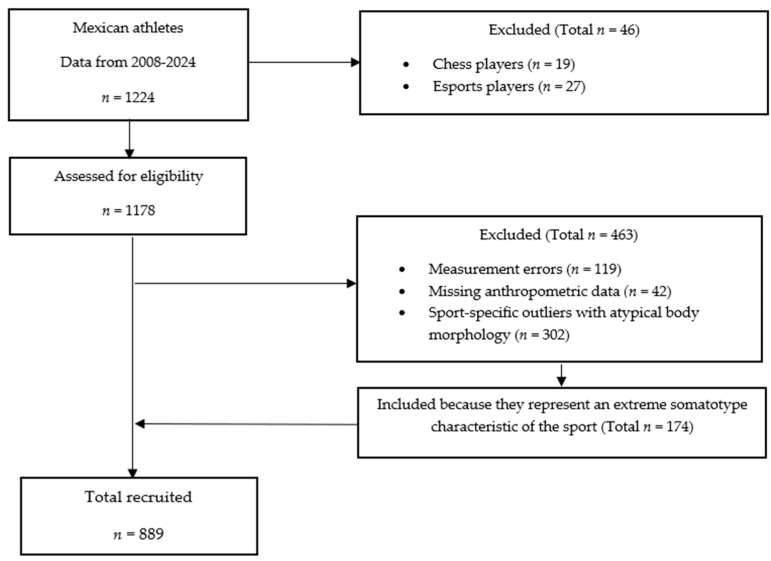
Flow diagram for Mexican athlete eligibility criteria.

Out of 43 sports disciplines, 1224 Mexican athletes agreed to participate and completed the anthropometric assessment. Two disciplines were excluded: chess (*n* = 11 male players; *n* = 8 female players) and esports (*n* = 27 male players), as they do not involve physical-athletic demands suitable for morphological evaluation. Consequently, 1178 athletes proceeded to the selection process; of these, 42 were excluded due to incomplete records for the analyzed variables, and 119 were removed due to measurement errors confirmed through dispersion analysis. A total of 302 outliers were identified using the interquartile range (IQR) method described in the Materials and Methods section, of which 174 were retained, as they represented extreme somatotypes characteristic of their respective sports disciplines (American football, powerlifting, and volleyball). The final sample consisted of 889 Mexican athletes (477 males and 412 females) (Figure 1).

### 3.2. Descriptive Data and Main Results

A total of 889 Mexican athletes (477 males and 412 females) from 43 sports disciplines across northern, central, and southern Mexico were evaluated. Table 1 shows the mean and standard deviation of body composition parameters and somatotype components by discipline and, when applicable, by playing position for male athletes. Similarly, Table 2 shows the corresponding data for female athletes.

**Figure 2:**
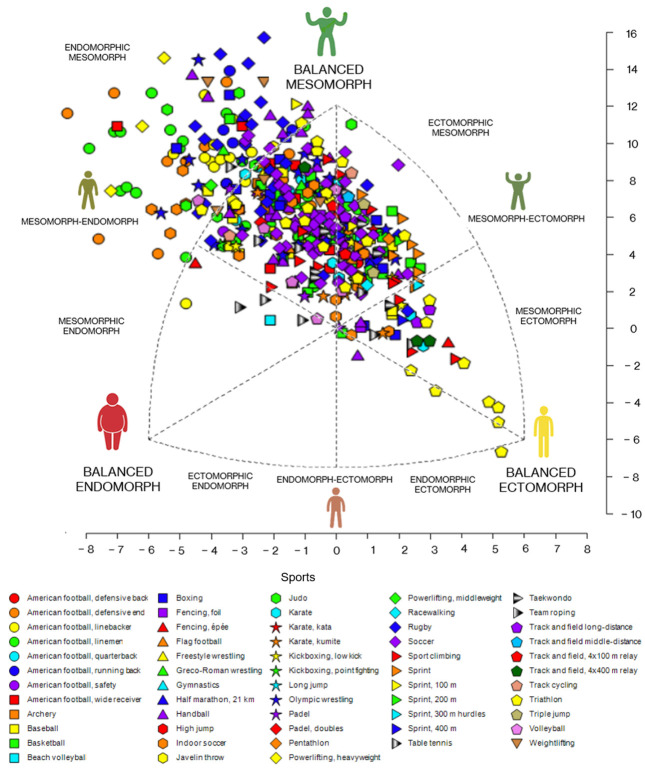
Somatotype of Mexican male athletes by sport (*n* = 477).

Among male athletes, the predominant somatotype was endomorphic mesomorph (52.4%), followed by balanced mesomorph (17.6%) and ectomorphic mesomorph (13.6%). Differences were found between somatotype categories (*p* < 0.001). However, no difference was observed between the proportions of balanced mesomorph and ectomorphic mesomorph (*p* = 0.236). Other somatotypes included mesomorph-endomorph (4.6%), mesomorphic ectomorph (4.2%), mesomorph-ectomorph (3.1%), central (1.9%), mesomorphic endomorph (1.3%), balanced ectomorph (1.0%), and balanced endomorph (0.2%) (Figure 2).

**Figure 3:**
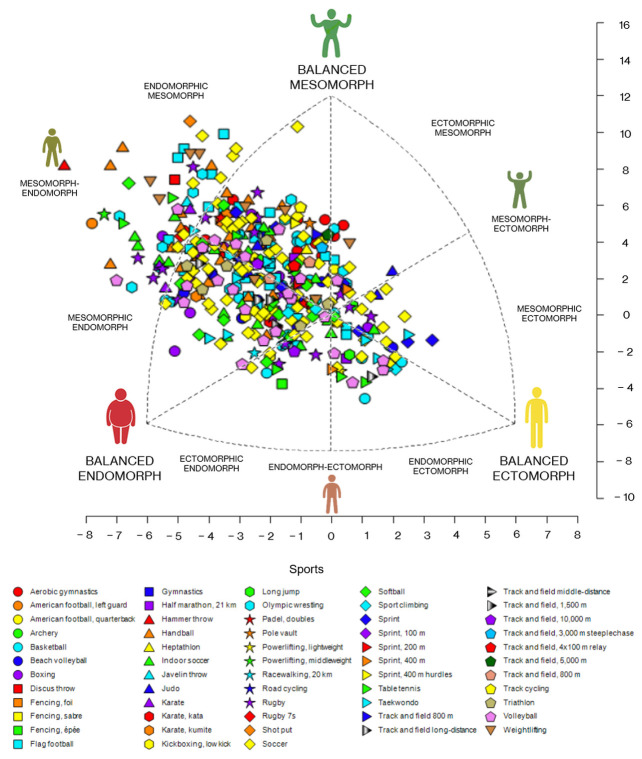
Somatotype of Mexican female athletes by sport (*n* = 412).

Among female athletes, no differences were found between somatotype categories (*p* = 0.514). The most frequently reported somatotypes were endomorphic mesomorph (24.5%), mesomorphic endomorph (24.0%), and mesomorph-endomorph (21.4%). Other observed somatotypes included central (8.0%), balanced endomorph (6.1%), balanced mesomorph (5.6%), balanced ectomorph (3.6%), endomorphic ectomorph (2.4%), endomorph-ectomorph (1.5%), mesomorphic ectomorph (1.2%), mesomorph-ectomorph (1.0%), ectomorphic mesomorph (0.5%), and ectomorphic endomorph (0.2%) (Figure 3).

### 3.3. Other Analyses

Athletes were divided into six macro-categories to group sports by sex (Table 3 and Table 4). Among male athletes (Table 3), differences were observed across all analyzed variables. Team sports exhibited the highest values for body mass (79.6 kg), height (178.0 cm), and body fat percentage (12.5%) compared to the other macro-categories (*p* < 0.001). The highest mesomorphy values were recorded in track and field (6.2), while the highest ectomorphy values were observed in sprint events (3.0). Endomorphy was greater in team sports (2.9) and combat sports (2.9) (*p* < 0.001).

Among female athletes (Table 4), differences between sport macro-categories were also identified. Athletes in track and field showed higher values for body mass (81.2 kg), height (164.8 cm), and body fat percentage (31.9%) (*p* < 0.001). The highest ectomorphy values were recorded in endurance events (2.3) (*p* = 0.002), while both mesomorphy and endomorphy were greater in track and field (*p* = 0.002; 7.0 and 5.0, respectively).

Figures S1 and S2 (Supplementary Materials) present somatocharts with athletes grouped into six macro-categories structured to facilitate the interpretation of the most predominant somatotypes among athletes. Figure S1 displays the somatochart for male athletes. In Figure S1a, corresponding to team sports, a predominance of the endomorphic mesomorph somatotype is observed (*p* < 0.001). The same somatotype also predominates in combat sports (*p* < 0.001) (Figure S1b). However, no predominant somatotype was found in individual sports (*p* = 0.056) (Figure S1c), endurance events (*p* = 0.718) (Figure S1d), or sprint events (*p* = 0.544) (Figure S1e). In track and field, only two athletes were reported, with no significance (*p* = 1.000) (Figure S1f).

Figure S2 illustrates the somatochart for female athletes, organized by macro-category and further subdivided by sport. No differences were found across any of the macro-categories among female athletes (*p* = 0.050).

## 4. Discussion

### 4.1. Key Results

The primary objective of the current study was to determine the somatotype of Mexican athletes by sex. Among male athletes, the predominant somatotype was endomorphic mesomorph (52.4%), followed by balanced mesomorph (17.6%) and ectomorphic mesomorph (13.6%), with differences between somatotype classifications. Among female athletes, the most frequently reported somatotypes were endomorphic mesomorph (24.5%), mesomorphic endomorph (24.0%), and mesomorph-endomorph (21.4%), with no differences between somatotype classifications. Male athletes exhibited greater morphological variability, whereas female athletes showed a more homogeneous distribution of morphological characteristics. Similar patterns have been reported in male international athletes [20,52,53]. However, studies on female athletes from other countries have reported a more centrally distributed somatotype with greater variability across disciplines [54,55,56]. This difference suggests that Mexican female athletes may exhibit lower morphological variability across disciplines, potentially influenced by ethnic and sociocultural factors or a generalized approach to their athletic training.

The second objective was to compare somatotype and body composition among sport macro-categories. In male athletes, differences were observed among all variables analyzed; team sports exhibited higher values for body mass (79.6 kg), height (178.0 cm), and %BF (12.5%). The highest mesomorphy was recorded in track and field (6.2), while the highest ectomorphy appeared in sprint events (3.0). Among female athletes, differences were also observed (*p* < 0.05); track and field athletes displayed higher body mass (81.2 kg), height (164.8 cm), and %BF (31.9%). Ectomorphy was highest in endurance events (2.3), while both mesomorphy and endomorphy reached their highest values in track and field (7.0 and 5.0, respectively). Sport macro-categories reflected specific morphological profiles: higher mesomorphy and endomorphy in team and combat sports, and higher ectomorphy in sprint and endurance events. In female athletes, high %BF and mesomorphy were observed in track and field. Grouping athletes by macro-categories facilitates the interpretation of somatotype patterns by highlighting the most suitable body prototypes for performance in each category. For example, Baranauskas et al. [6] reported higher endomorphy and mesomorphy in athletes from combat sports, while Gutnik et al. [57] and Campa et al. [58] described distinct profiles in team sports with higher mesomorphy in basketball players and higher ectomorphy in soccer players. These findings support the idea that somatotype reflects both training adaptations and the morphological demands inherent to each sport modality, a pattern clearly observed in the context of Mexican athletes.

Overall, the results of this study demonstrate that somatotype varies according to sex and type of sport, reflecting both functional adaptations and structural requirements related to body physique. These findings may be helpful for talent identification, designing individualized training programs, and implementing sport-specific nutritional strategies. Moreover, they provide a solid scientific foundation for the monitoring and physical development of high-performance athletes in Mexico. Higher mesomorphy is consistently associated with superior strength, power, and explosive performance, as seen in exercises such as the bench press, back squat, vertical jump, and sprinting. In contrast, higher ectomorphy tends to favor flexibility, aerobic capacity, and endurance, but may negatively impact strength and power outputs. Conversely, higher endomorphy generally predicts poorer performance in explosive and aerobic tasks, but may be advantageous in some strength-related activities. These relationships are evident across a range of sports and age groups, and somatotype can explain a significant portion of variance in physical fitness and sport-specific skills, making it a valuable consideration for talent identification and individualized training programs [7,17,23,59,60].

### 4.2. Strengths and Limitations

This study provides valuable insights into the somatotypes of Mexican athletes across various sports disciplines; however, it also reveals several limitations. First, the sample was obtained through purposive sampling and included only athletes who voluntarily agreed to participate, with representation limited to specific regions of Mexico. Second, for some sports disciplines, data were available for only a single subject. This limited representation constrains the generalizability of the findings within those particular disciplines. This limitation is primarily due to the logistical and structural challenges of recruiting athletes across multiple competitive modalities in a national context. Consequently, results in these disciplines should be interpreted with caution. These constraints reinforce the exploratory nature of the study and highlight the need for further research with larger and more balanced samples. Moreover, the sample encompassed a heterogeneous range of competitive levels, including athletes from regional competitions to world-class performers. Such variability may have attenuated the somatotype differences typically observed at specific elite levels, thereby introducing a bias toward intermediate and less sport-specific profiles. The magnitude of this potential bias may be moderate, particularly in disciplines where performance is closely linked to body morphology, as elite athletes tend to exhibit superior proprioceptive capacities, initiate training earlier, and engage in more intensive training regimens compared to their lower-level counterparts [61,62].

### 4.3. Interpretation

The findings of this study provide a robust characterization of somatotype and body composition among Mexican athletes, identifying relevant differences by sex and sports disciplines. These differences reflect not only physiological adaptations to training but also specific morphological demands inherent to each sport modality, consistent with findings reported in international studies, particularly among male athletes [20,52,53]. The data obtained represent a valuable foundation for understanding the predominant body profiles among Mexican athletes. They may contribute to the development of tailored strategies for assessment, selection, and planning within the national sports context.

Carter [16] demonstrated differences in somatotype components, particularly endomorphy, among male athletes practicing the same sport, such as weightlifters and wrestlers, from different nationalities. Similarly, the visual trends observed in the somatocharts of both male and female Mexican athletes align with those reported by Carter and Heath [63]. Ethnic group differences are evident, suggesting that the use of global averages based on international competitions or events [20,64] may introduce bias into data interpretation. Although regional or national variations are occasionally observed, current evidence indicates that somatotype distribution is more strongly influenced by factors such as genetics, biological maturation, training, environment, and nutrition. At the same time, nationality acts as an indirect and not necessarily determinant factor [6,57,65,66].

To the best of our knowledge, only one study conducted among Lithuanian athletes has compared somatotypes across multiple sports disciplines at the national level. The findings revealed that each sport favored a distinct body type: kayakers were predominantly endomorphic, basketball players presented an endomorphic-mesomorphic profile, and soccer players exhibited a more ectomorphic build [57]. It is important to note that somatotype is influenced by both training-induced adaptations and self-selection into sports. Individuals may be drawn to disciplines that match their natural physique, such as leaner athletes choosing endurance running or more muscular individuals gravitating toward strength and power sports. Therefore, somatotype should be understood as the result of a dynamic interaction between biological predisposition and the specific demands of sport participation.

Although somatotype may serve as a valuable tool for identifying athletic potential, it should not be considered in isolation from other factors. Successful athletes are also distinguished by psychological attributes such as self-confidence, motivation, and resilience, as well as by their sport-specific experience, technical skills, and environmental support [67,68,69]. These elements, when considered alongside physical profile, are critical for the long-term development and maintenance of athletic performance.

Current research strongly supports emphasizing in the discussion that anthropometric data, such as somatotype, should not be used as standalone criteria for talent identification. While anthropometric and physical performance measures can help distinguish between competitive levels and contribute to early talent identification, their predictive power is limited when used in isolation. Multiple studies highlight that talent identification is a complex, multidimensional process influenced by technical, tactical, psychological, and sociological factors in addition to physical attributes. Relying solely on anthropometric data risks overlooking late-maturing or otherwise talented individuals who may excel in other critical domains. Therefore, integrating anthropometric data within a broader, holistic assessment framework is necessary to improve the accuracy and fairness of talent identification and development programs [70,71,72,73,74].

Moreover, it is essential to recognize that anthropometric and somatotype profiles can be influenced by training phases within the competitive cycle. Recent evidence highlights that variations in preparation and competition periods can affect both body composition and performance-related parameters, underscoring the dynamic nature of morphological characteristics [75].

### 4.4. Generalizability

A wide variety of disciplines were included in the study. However, representation by sport was limited in some cases. This was partly influenced by the popularity of certain sports practiced in Mexico and those that receive greater institutional support. Such limitations may reduce the applicability of the findings to disciplines with lower representation. Therefore, the results may be beneficial for national sports contexts or countries with similar competitive structures and anthropometric characteristics. Future multicenter studies at the national level are recommended to strengthen the external validity of these findings.

## 5. Conclusions

This study provided a detailed characterization of somatotypes in Mexican athletes, revealing specific patterns by sex and sport macro-categories. In a sample of 477 male and 412 female athletes, the most frequent somatotypes among males were endomorphic mesomorph, balanced mesomorph, and ectomorphic mesomorph, with differences among somatotype classifications, reflecting a predominance of traits associated with strength and power. Among females, the reported somatotypes were endomorphic mesomorph, mesomorphic endomorph, and mesomorph-endomorph, with no differences among categories, suggesting a relatively higher proportion of adiposity. Compared to international athletes, Mexican female athletes exhibited a more pronounced endomorphic component. Additionally, the macro-categorical groupings revealed somatotype differences in both males and females, reflecting distinct morphological profiles according to sport type.

These findings underscore the value of somatotyping as a strategic tool for talent identification, training planning, and the personalization of nutritional interventions, particularly in disciplines with specific physical demands. It is recommended that somatotype assessment be incorporated into regular monitoring protocols in national sports centers and that these morphological profiles be considered in physical preparation programs to optimize performance and reduce injury risk. Likewise, an individualized approach is advised, considering sex, sports discipline, and competitive level. This study provides novel evidence of somatotypes in Mexican athletes, contributing to a field that has historically lacked comprehensive research in this population. Future research is encouraged to validate personalized interventions based on somatotype, explore its relationship with injury risk and training adaptation, and include comparisons across different levels of elite competition. The development of longitudinal studies is also recommended to assess the evolution of somatotype profiles in Mexican athletes and their long-term impact on athletic performance.

## Figures and Tables

**Table 1:** Descriptive characteristics and somatotype ratings of male athletes by sport.

Sport	*n*	Age	Body Mass (kg)	Height (cm)	BMI (kg/m^2^)	%BF (Lean et al.) [45]	ENDO	MESO	ECTO
American football, defensive back	1	22.7	76.4	182.0	23.1	9.9	2.2	4.3	2.8
American football, defensive end	14	22.8 ± 1.6	110.4 ± 16.2	184.8 ± 3.5	32.4 ± 5.3	21.6 ± 10.9	4.9 ± 1.9	7.0 ± 1.6	0.7 ± 0.6
American football, linemen	31	22.3 ± 1.6	94.3 ± 22.7	178.6 ± 5.8	29.4 ± 5.8	16.6 ± 7.4	4.1 ± 1.9	6.4 ± 1.5	1.1 ± 0.8
American football, linebacker	15	22.2 ± 2.1	93.7 ± 6.1	178.9 ± 4.5	29.2 ± 1.4	15.7 ± 4.8	4.2 ± 0.8	6.8 ± 1.0	0.6 ± 0.4
American football, quarterback	5	22.5 ± 1.9	93.6 ± 7.5	184.8 ± 4.0	27.4 ± 1.4	15.0 ± 4.4	3.3 ± 0.8	6.2 ± 0.3	1.3 ± 0.3
American football, running back	11	22.0 ± 1.9	80.6 ± 9.2	171.2 ± 6.4	27.5 ± 2.3	12.2 ± 3.0	3.2 ± 0.7	7.0 ± 0.9	0.8 ± 0.6
American football, safety	1	20.8	87.4	177.0	27.9	17.1	4.4	5.3	0.8
American football, wide receiver	11	23.3 ± 1.6	85.0 ± 16.1	178.7 ± 5.5	26.6 ± 4.4	13.7 ± 5.7	3.1 ± 1.5	5.7 ± 1.5	1.5 ± 0.9
Archery	1	21.0	89.1	180.0	27.5	14.8	3.5	6.2	1.0
Baseball	1	21.0	71.5	170.0	24.7	15.2	3.2	6.6	1.4
Baseball, catcher	1	21.0	92.7	182.0	28.0	17.2	4.3	5.6	1.0
Baseball, center fielder	1	19.0	59.7	168.0	21.2	7.3	1.8	4.8	2.9
Baseball, infielder	1	17.0	64.8	175.0	21.2	6.7	1.5	4.6	3.3
Baseball, pitcher	8	19.6 ± 1.9	77.9 ± 9.5	177.9 ± 7.5	24.6 ± 2.7	15.9 ± 4.9	3.5 ± 1.0	4.6 ± 1.0	2.0 ± 1.2
Baseball, second baseman	1	23.0	76.5	173.0	25.6	13.4	2.9	6.4	1.3
Basketball	25	20.9 ± 1.2	87.0 ± 10.1	186.5 ± 6.9	25.0 ± 2.2	11.5 ± 3.9	2.6 ± 0.9	5.1 ± 0.9	2.3 ± 0.9
Basketball, center	2	20.5 ± 0.7	83.9 ± 1.3	190.0 ± 1.4	23.2 ± 0.0	7.5 ± 0.7	1.8 ± 0.1	4.3 ± 0.4	3.1 ± 0.1
Basketball, forward	4	19.5 ± 0.6	79.2 ± 6.4	185.0 ± 3.5	23.2 ± 1.3	9.4 ± 2.2	2.3 ± 0.7	4.4 ± 0.8	3.0 ± 0.6
Basketball, point guard	3	19.7 ± 3.8	74.1 ± 3.0	177.0 ± 4.4	23.6 ± 0.6	9.5 ± 0.4	2.1 ± 0.1	4.9 ± 0.8	2.3 ± 0.5
Beach volleyball	3	20.3 ± 1.5	77.5 ± 14.8	184.0 ± 6.1	22.8 ± 2.9	11.7 ± 6.0	2.8 ± 1.3	4.0 ± 0.9	3.1 ± 1.0
Boxing	14	20.4 ± 2.0	70.4 ± 11.9	171.4 ± 6.9	23.9 ± 3.3	12.2 ± 3.1	2.9 ± 1.0	5.6 ± 1.6	2.0 ± 1.3
Boxing < 63 kg	1	22.0	64.3	174.0	21.2	11.7	2.1	4.6	3.2
Boxing < 69 kg	1	19.0	70.2	174.0	23.2	9.4	2.0	4.5	2.3
Boxing < 75 kg	1	18.0	76.9	175.0	25.1	14.7	3.3	5.8	1.5
Boxing > 91 kg	1	19.0	94.3	184.0	27.9	15.5	4.4	6.1	1.1
Fencing, épée	2	18.5 ± 0.7	73.4 ± 32.7	178.0 ± 12.7	22.6 ± 7.1	15.6 ± 14.8	3.5 ± 3.2	4.0 ± 1.8	3.1 ± 2.5
Fencing, foil	3	20.7 ± 2.1	62.0 ± 5.5	167.7 ± 2.1	22.1 ± 2.4	12.6 ± 1.7	3.1 ± 0.4	4.8 ± 1.1	2.5 ± 1.2
Flag football	4	22.2 ± 1.5	81.8 ± 5.2	182.0 ± 4.1	24.6 ± 0.9	13.6 ± 2.4	2.8 ± 0.4	4.8 ± 0.4	2.1 ± 0.4
Freestyle wrestling	1	21.0	91.8	182.0	27.7	13.1	4.1	5.9	1.0
Freestyle wrestling < 74 kg	1	24.0	75.7	168.0	26.8	12.3	3.0	6.2	0.8
Greco-Roman wrestling	1	17.0	68.1	167.0	24.4	9.7	2.7	5.4	1.4
Greco-Roman wrestling < 60 kg	1	21.0	63.2	162.0	24.1	10.1	2.3	5.7	1.2
Greco-Roman wrestling < 63 kg	1	21.0	64.7	167.0	23.2	7.6	1.9	5.7	1.9
Greco-Roman wrestling < 82 kg	1	23.0	83.7	169.0	29.3	14.1	3.9	6.9	0.3
Gymnastics	1	21.0	62.5	166.0	22.7	7.3	2.1	6.1	2.0
Half marathon, 21 km	2	21.0 ± 1.4	72.7 ± 12.2	177.0 ± 5.7	23.1 ± 2.4	9.2 ± 2.5	2.3 ± 0.6	4.5 ± 0.3	2.5 ± 0.8
Handball	14	20.1 ± 1.1	76.9 ± 10.9	175.5 ± 6.8	24.9 ± 2.9	10.7 ± 2.8	2.9 ± 0.9	6.0 ± 1.6	1.8 ± 1.1
Handball, back	1	23.0	93.2	184.0	27.5	17.0	3.1	6.2	1.2
Handball, center	3	19.7 ± 1.5	70.8 ± 11.0	170.3 ± 3.8	24.3 ± 2.9	13.0 ± 2.8	2.9 ± 0.7	5.5 ± 2.0	1.7 ± 0.9
Handball, goalkeeper	4	21.2 ± 1.0	81.4 ± 12.4	180.8 ± 5.2	24.9 ± 3.0	16.3 ± 3.0	3.4 ± 0.6	5.0 ± 1.1	2.1 ± 1.2
Handball, left back	1	18.0	78.6	186.0	22.7	9.3	2.2	4.7	3.2
Handball, left wing	1	21.0	70.2	164.0	26.1	6.8	1.7	7.1	0.8
Handball, line player	1	23.0	93.5	174.0	30.9	16.6	4.3	8.5	0.1
Handball, right back	2	20.5 ± 3.5	88.0 ± 0.0	182.5 ± 0.7	26.6 ± 0.0	16.8 ± 2.0	3.4 ± 0.2	6.0 ± 0.3	1.4 ± 0.1
Handball, right wing	2	21.5 ± 0.7	69.8 ± 2.0	173.5 ± 0.7	23.2 ± 0.4	11.6 ± 0.9	2.7 ± 0.0	5.0 ± 0.1	2.3 ± 0.1
Handball, wing	1	22.0	71.2	165.0	26.2	8.4	1.9	6.7	0.8
High jump	1	20.4	70.0	178.4	22.1	12.1	2.3	4.1	3.1
Indoor soccer	5	20.4 ± 2.6	71.3 ± 17.8	170.1 ± 9.9	24.3 ± 3.9	14.0 ± 5.5	3.5 ± 1.5	5.3 ± 0.9	1.8 ± 1.0
Indoor soccer, defender	5	19.8 ± 3.0	79.8 ± 14.0	176.0 ± 4.3	25.8 ± 4.6	15.4 ± 8.4	3.8 ± 1.8	5.0 ± 1.7	1.7 ± 1.6
Indoor soccer, forward	1	18.0	83.8	175.0	27.4	12.2	4.0	5.7	0.9
Indoor soccer, goalkeeper	4	21.0 ± 1.8	69.7 ± 9.5	174.2 ± 7.8	22.9 ± 2.8	16.1 ± 8.6	3.6 ± 1.8	4.2 ± 1.4	2.5 ± 1.2
Indoor soccer, midfielder	5	20.2 ± 1.6	70.2 ± 5.2	170.4 ± 5.2	24.2 ± 2.3	14.8 ± 4.5	3.5 ± 1.1	5.6 ± 1.1	1.8 ± 1.0
Javelin throw	2	19.0 ± 1.4	79.2 ± 8.8	175.0 ± 0.0	25.9 ± 2.9	11.7 ± 1.8	2.5 ± 0.3	6.2 ± 0.7	1.4 ± 0.9
Judo	7	19.6 ± 2.1	65.2 ± 8.5	167.0 ± 6.3	23.4 ± 2.3	11.6 ± 5.3	2.9 ± 1.4	5.7 ± 1.2	1.9 ± 0.8
Judo < 100 kg	1	18.0	97.2	170.0	33.6	17.3	5.5	8.8	0.1
Judo < 55 kg	1	20.0	53.5	164.0	19.9	11.2	2.6	4.3	3.3
Judo < 73 kg	1	19.0	73.6	167.0	26.4	15.9	4.7	5.8	0.8
Judo < 81 kg	1	17.0	81.2	164.0	30.2	10.3	3.2	8.1	0.1
Karate	4	19.5 ± 1.7	69.5 ± 14.9	174.8 ± 6.0	22.6 ± 4.1	12.2 ± 4.6	2.8 ± 0.7	4.5 ± 1.5	2.8 ± 1.6
Karate, kata	2	17.0 ± 0.0	56.4 ± 3.8	162.5 ± 0.7	21.4 ± 1.2	7.2 ± 2.4	1.9 ± 0.3	5.1 ± 0.1	2.5 ± 0.6
Karate, kumite	6	19.8 ± 1.5	72.3 ± 7.0	174.8 ± 5.2	23.7 ± 2.3	14.1 ± 3.9	3.2 ± 0.7	4.9 ± 1.6	2.2 ± 1.1
Kickboxing, low kick	6	19.7 ± 2.0	64.6 ± 7.6	171.2 ± 4.0	22.1 ± 2.4	10.9 ± 3.4	2.6 ± 0.9	4.4 ± 0.5	2.7 ± 1.1
Kickboxing, point fighting	2	18.0 ± 1.4	76.6 ± 10.6	175.0 ± 7.1	24.9 ± 1.5	15.9 ± 7.8	4.5 ± 0.9	5.5 ± 0.1	1.6 ± 0.1
Long jump	1	20.0	70.1	173.0	23.4	10.4	2.5	5.3	2.1
Olympic wrestling	19	20.9 ± 1.4	73.0 ± 13.9	170.0 ± 6.7	25.1 ± 3.4	11.9 ± 4.4	3.2 ± 1.3	5.9 ± 1.2	1.5 ± 0.9
Olympic wrestling < 65 kg	1	19.0	66.3	168.0	23.5	9.9	3.1	5.4	1.8
Padel	4	21.2 ± 1.7	74.2 ± 10.0	175.0 ± 2.5	24.2 ± 3.2	15.3 ± 1.0	3.5 ± 0.4	4.3 ± 1.3	2.1 ± 1.1
Padel, doubles	1	19.0	77.6	176.0	25.1	18.1	4.0	5.7	1.6
Pentathlon	1	23.5	76.2	184.5	22.5	6.8	1.6	4.9	3.3
Powerlifting < 90 kg	1	25.0	80.1	170.0	27.7	17.8	4.7	6.5	0.6
Powerlifting < 125 kg	1	32.0	128.4	179.0	40.1	34.7	5.6	10.2	0.1
Powerlifting < 100 kg	1	31.0	100.3	166.0	36.4	26.4	6.3	8.7	0.1
Powerlifting < 140 kg	1	18.0	131.6	190.0	36.5	27.7	7.3	7.5	0.1
Racewalking	1	19.0	59.5	168.0	21.1	7.4	2.0	3.6	2.9
Rugby	16	19.9 ± 1.9	78.6 ± 9.4	172.6 ± 4.8	26.5 ± 3.2	14.5 ± 6.5	3.6 ± 1.2	6.5 ± 1.5	1.3 ± 1.1
Rugby, center	2	21.0 ± 1.4	77.5 ± 3.6	174.0 ± 2.8	25.6 ± 0.3	16.9 ± 0.9	3.4 ± 0.1	6.0 ± 0.3	1.3 ± 0.0
Rugby, fly-half	1	20.0	63.1	170.0	21.8	12.1	3.0	4.3	2.7
Rugby, hooker	3	20.0 ± 1.0	84.6 ± 7.3	168.3 ± 2.1	29.8 ± 1.9	12.6 ± 4.2	3.6 ± 1.1	8.7 ± 0.9	0.2 ± 0.2
Rugby, prop	2	22.0 ± 1.4	83.8 ± 5.2	176.2 ± 3.9	27.1 ± 0.3	13.2 ± 4.8	2.9 ± 0.3	7.0 ± 0.8	1.0 ± 0.0
Rugby, scrum-half	1	19.0	74.0	180.0	22.8	5.3	1.5	4.0	2.8
Rugby, wing	1	21.0	66.2	171.0	22.6	5.1	1.4	5.8	2.4
Soccer	43	21.2 ± 1.7	73.6 ± 7.2	175.9 ± 5.3	23.8 ± 1.6	11.6 ± 3.5	2.6 ± 0.8	5.2 ± 0.8	2.2 ± 0.7
Soccer, defender	6	21.0 ± 2.0	72.8 ± 5.0	173.7 ± 5.7	24.2 ± 2.2	11.0 ± 3.1	2.5 ± 0.5	5.7 ± 1.2	1.9 ± 1.1
Soccer, forward	7	20.3 ± 1.8	68.0 ± 11.0	170.3 ± 8.0	23.3 ± 1.9	9.1 ± 3.2	2.5 ± 0.7	5.3 ± 0.7	2.1 ± 0.5
Soccer, goalkeeper	1	20.0	81.7	173.0	27.3	12.7	3.7	7.4	0.8
Soccer, midfielder	6	21.0 ± 1.3	68.0 ± 9.7	171.9 ± 7.9	22.9 ± 1.7	10.3 ± 2.9	2.4 ± 0.6	5.3 ± 0.9	2.3 ± 0.7
Sport climbing	13	19.5 ± 1.3	62.9 ± 7.4	170.8 ± 6.7	21.5 ± 2.1	9.6 ± 4.6	2.3 ± 0.8	4.5 ± 1.2	2.9 ± 1.2
Sprint	9	20.8 ± 1.7	70.6 ± 9.4	179.5 ± 8.3	21.8 ± 1.4	5.6 ± 0.9	1.3 ± 0.3	4.4 ± 0.5	3.3 ± 0.7
Sprint, 100 m	3	22.3 ± 0.6	76.0 ± 9.9	173.3 ± 2.9	25.2 ± 2.5	8.1 ± 0.4	2.0 ± 0.2	6.3 ± 1.4	1.5 ± 0.8
Sprint, 200 m	1	23.0	73.5	181.0	22.4	5.6	1.1	4.5	3.0
Sprint, 300 m hurdles	1	22.0	59.7	174.0	19.7	6.7	1.4	3.9	4.0
Sprint, 400 m	2	20.0 ± 2.8	77.3 ± 6.7	186.0 ± 2.8	22.4 ± 2.6	8.8 ± 0.6	2.3 ± 0.0	4.2 ± 0.9	3.4 ± 1.4
Table tennis	9	19.8 ± 1.4	69.5 ± 5.9	173.8 ± 4.4	23.1 ± 2.4	12.0 ± 4.6	3.1 ± 1.0	4.2 ± 1.4	2.5 ± 1.1
Taekwondo	6	18.3 ± 1.4	67.4 ± 8.4	174.2 ± 4.8	22.1 ± 1.7	9.6 ± 3.5	2.4 ± 0.8	4.6 ± 0.7	2.8 ± 0.6
Taekwondo < 74 kg	1	24.0	71.9	174.0	23.7	14.0	2.5	4.9	2.1
Team roping, heeler	1	21.0	82.4	184.5	24.3	26.1	5.5	4.6	2.5
Track and field, 4 × 100 m relay	2	20.5 ± 2.1	72.8 ± 5.6	175.0 ± 1.4	23.8 ± 1.5	8.9 ± 0.8	2.0 ± 0.3	4.8 ± 0.5	2.1 ± 0.6
Track and field, 4 × 400 m relay	3	22.0 ± 2.6	74.0 ± 7.1	182.7 ± 7.1	22.3 ± 3.7	8.5 ± 1.2	1.8 ± 0.2	3.9 ± 1.9	3.4 ± 2.0
Track and field long-distance	3	22.4 ± 2.9	58.9 ± 4.4	171.1 ± 2.5	20.2 ± 2.1	7.8 ± 2.4	1.6 ± 0.3	3.9 ± 1.0	3.6 ± 1.2
Track and field middle-distance	1	21.0	59.1	164.0	22.0	7.3	1.8	5.6	2.2
Track cycling	2	20.0 ± 1.4	80.2 ± 10.0	175.1 ± 9.8	26.1 ± 0.3	11.2 ± 8.7	2.6 ± 2.5	5.3 ± 0.1	1.2 ± 0.3
Triathlon	6	19.8 ± 1.3	69.5 ± 6.8	171.7 ± 6.5	23.6 ± 2.1	15.6 ± 5.2	3.5 ± 1.1	4.9 ± 1.1	2.1 ± 0.9
Triple jump	2	22.5 ± 0.7	79.0 ± 1.8	183.9 ± 4.1	23.3 ± 0.6	10.6 ± 2.1	1.7 ± 0.3	4.8 ± 0.9	2.8 ± 0.4
Volleyball	12	20.7 ± 1.8	75.7 ± 10.2	186.3 ± 10.7	22.0 ± 3.5	9.7 ± 4.1	2.3 ± 1.3	3.8 ± 1.9	3.8 ± 2.1
Volleyball, center	1	19.0	73.5	200.0	18.4	6.1	1.2	1.3	6.4
Volleyball, libero	2	21.0 ± 2.8	69.2 ± 7.0	169.5 ± 3.5	24.0 ± 1.4	8.9 ± 3.7	2.4 ± 0.9	5.8 ± 0.9	1.6 ± 0.3
Volleyball, middle blocker	4	21.0 ± 0.8	91.6 ± 17.4	190.8 ± 4.9	25.1 ± 4.7	14.6 ± 2.9	3.4 ± 1.1	4.6 ± 1.9	2.6 ± 2.0
Volleyball, opposite hitter	1	23.0	85.9	200.0	21.5	12.9	2.2	2.3	4.6
Volleyball, outside hitter	5	22.4 ± 1.1	81.5 ± 9.6	179.7 ± 2.6	25.2 ± 2.5	10.7 ± 3.4	2.1 ± 0.7	6.2 ± 0.9	1.9 ± 1.0
Volleyball, setter	1	21.0	71.3	171.0	24.4	5.2	1.9	5.5	1.6
Weightlifting	2	20.0 ± 0.0	83.7 ± 8.0	171.5 ± 7.8	28.4 ± 0.1	13.1 ± 6.2	3.6 ± 1.3	7.0 ± 1.7	0.6 ± 0.2
Weightlifting < 67 kg	1	18.0	68.3	169.0	23.9	6.6	2.1	5.4	1.7
Weightlifting < 81 kg	1	19.0	80.8	175.0	26.4	12.2	3.4	6.3	1.1
Weightlifting < 89 kg	1	20.0	89.4	167.0	32.1	9.9	4.2	8.9	0.1
TOTAL	477								

Note. This table shows the sample size (*n*), the mean and standard deviation of the somatotype components (endomorphy, mesomorphy, and ectomorphy) according to the Heath–Carter method, body mass (kg), height (cm), body mass index (BMI, kg/m^2^), and body fat percentage estimated using Equation (2) proposed by Lean et al. [45].

**Table 2:** Descriptive characteristics and somatotype ratings of female athletes by sport.

Sport	*n*	Age	Body Mass (kg)	Height (cm)	BMI (kg/m^2^)	%BF (Lean et al.) [45]	ENDO	MESO	ECTO
Aerobic gymnastics	11	20.2 ± 2.4	56.4 ± 4.2	158.7 ± 5.3	22.4 ± 1.7	23.0 ± 2.9	3.5 ± 1.1	4.9 ± 0.5	1.8 ± 0.8
American football, left guard	1	22.0	89.2	168.1	31.6	37.9	7.9	6.6	0.1
American football, quarterback	1	24.0	62.4	169.6	21.8	23.1	2.6	4.0	2.7
Archery	1	17.0	44.0	159.0	17.4	16.1	2.5	2.7	4.4
Basketball	22	20.4 ± 1.8	69.0 ± 12.6	172.6 ± 7.6	22.8 ± 2.9	24.4 ± 4.0	3.8 ± 1.3	3.6 ± 1.0	2.4 ± 1.0
Basketball, center	2	22.0 ± 0.0	78.8 ± 0.8	183.0 ± 0.0	23.6 ± 0.2	27.8 ± 0.5	4.5 ± 0.2	2.4 ± 0.4	2.6 ± 0.1
Basketball, forward	1	25.0	66.3	167.0	23.8	27.0	3.6	4.0	1.6
Basketball, point guard	1	20.0	56.2	160.0	22.0	22.8	3.6	3.6	2.0
Beach volleyball	4	20.5 ± 1.7	61.2 ± 7.1	166.8 ± 2.7	22.1 ± 2.6	22.8 ± 3.4	3.8 ± 1.7	3.4 ± 0.8	2.5 ± 1.3
Beach volleyball, all-round player	1	22.0	67.8	162.0	25.8	27.1	4.6	3.9	0.8
Beach volleyball, blocker	2	21.0 ± 0.0	67.2 ± 0.3	173.0 ± 0.0	22.5 ± 0.1	24.2 ± 0.1	3.6 ± 0.0	3.1 ± 0.6	2.5 ± 0.1
Beach volleyball, defender	3	21.3 ± 1.1	58.5 ± 4.1	163.3 ± 1.1	21.9 ± 1.2	23.4 ± 1.9	4.7 ± 0.4	3.2 ± 0.3	2.2 ± 0.5
Boxing	7	21.0 ± 1.6	60.2 ± 6.6	161.5 ± 4.0	23.0 ± 2.2	26.4 ± 3.9	4.8 ± 1.4	4.2 ± 1.2	1.7 ± 1.0
Discus throw	1	21.0	80.0	165.0	29.4	30.5	5.2	6.4	0.1
Fencing, foil	1	22.0	63.1	163.3	23.7	31.1	6.2	4.7	1.4
Fencing, sabre	1	19.0	55.5	161.0	21.4	24.3	4.3	3.5	2.3
Fencing, épée	1	18.0	67.9	178.0	21.4	25.8	5.0	2.3	3.4
Flag football	11	20.0 ± 2.0	58.1 ± 7.3	160.2 ± 6.5	22.7 ± 1.9	24.7 ± 2.5	4.1 ± 0.9	4.3 ± 1.2	1.8 ± 0.8
Flag football, cornerback	3	17.3 ± 1.5	50.3 ± 0.1	159.7 ± 2.3	19.8 ± 0.6	19.4 ± 1.1	2.8 ± 0.0	3.2 ± 0.9	3.1 ± 0.4
Flag football, quarterback	3	23.0 ± 0.0	67.6 ± 14.3	164.7 ± 8.1	24.7 ± 2.9	30.3 ± 3.6	5.6 ± 0.6	5.1 ± 0.5	1.3 ± 0.6
Flag football, safety	2	22.5 ± 0.7	70.8 ± 8.0	162.2 ± 3.2	26.9 ± 2.0	29.8 ± 4.7	5.0 ± 0.3	6.5 ± 1.0	0.6 ± 0.3
Flag football, wide receiver	10	19.7 ± 1.3	59.2 ± 4.4	157.9 ± 6.1	23.8 ± 1.9	25.8 ± 2.7	4.3 ± 0.5	5.1 ± 1.0	1.3 ± 0.8
Gymnastics	1	21.0	60.4	162.0	23.0	23.5	3.7	4.9	1.6
Half marathon, 21 km	2	20.5 ± 3.5	49.3 ± 3.2	157.9 ± 0.8	19.9 ± 1.5	21.4 ± 1.1	3.3 ± 0.4	3.4 ± 1.7	3.0 ± 0.8
Hammer throw	1	19.0	96.7	165.0	35.5	43.1	8.8	8.6	0.1
Handball	13	20.6 ± 2.0	64.2 ± 6.5	163.2 ± 5.7	24.1 ± 2.1	26.7 ± 4.2	4.7 ± 1.3	4.8 ± 1.4	1.4 ± 0.7
Handball, back	3	20.3 ± 0.6	60.9 ± 10.3	162.0 ± 11.4	23.0 ± 0.6	24.5 ± 1.2	4.1 ± 0.5	4.3 ± 0.6	1.6 ± 0.5
Handball, center	3	20.3 ± 0.6	56.7 ± 1.1	153.3 ± 4.9	24.2 ± 1.2	27.2 ± 3.1	4.8 ± 1.4	5.3 ± 0.3	0.9 ± 0.6
Handball, goalkeeper	2	21.5 ± 2.1	66.5 ± 11.0	164.5 ± 2.1	24.6 ± 4.7	29.8 ± 6.0	6.2 ± 1.9	4.2 ± 1.5	1.5 ± 1.7
Handball, lateral	1	19.0	69.2	165.0	25.4	26.0	3.7	5.5	1.0
Handball, left back	1	20.0	70.0	165.0	25.7	28.3	4.2	4.7	0.9
Handball, left wing	3	20.3 ± 2.1	63.8 ± 2.8	158.3 ± 4.6	25.4 ± 1.0	26.7 ± 1.3	5.0 ± 0.8	5.1 ± 0.6	0.7 ± 0.4
Handball, pivot	2	20.5 ± 0.7	73.0 ± 8.6	161.0 ± 1.4	28.1 ± 2.8	32.4 ± 4.2	5.7 ± 1.7	7.4 ± 1.0	0.3 ± 0.3
Handball, right wing	1	20.0	58.8	160.0	23.0	25.7	4.5	4.7	1.5
Handball, wing	1	17.0	63.6	162.7	23.9	27.7	5.6	5.3	1.3
Heptathlon	1	22.0	54.1	156.5	22.2	22.2	2.5	3.9	1.7
Indoor soccer	4	20.5 ± 1.0	59.4 ± 2.9	161.5 ± 1.3	22.8 ± 1.1	26.6 ± 3.1	4.6 ± 0.9	4.3 ± 0.8	1.7 ± 0.5
Indoor soccer, defender	4	20.8 ± 2.2	56.5 ± 3.4	161.5 ± 6.8	21.8 ± 2.3	23.9 ± 4.0	4.2 ± 1.6	4.0 ± 1.2	2.3 ± 1.3
Indoor soccer, forward	5	19.6 ± 1.5	53.3 ± 3.6	159.8 ± 3.8	20.8 ± 1.2	22.4 ± 2.7	3.6 ± 0.9	3.7 ± 1.0	2.5 ± 0.7
Indoor soccer, goalkeeper	4	20.0 ± 1.8	64.3 ± 1.6	158.8 ± 5.7	25.6 ± 1.4	30.2 ± 1.4	5.9 ± 0.7	4.9 ± 0.9	0.8 ± 0.7
Indoor soccer, midfielder	5	20.8 ± 2.2	53.9 ± 4.6	154.8 ± 4.1	22.6 ± 2.6	24.4 ± 3.5	4.9 ± 1.0	4.2 ± 1.2	1.6 ± 1.1
Javelin throw	1	22.0	76.2	163.5	28.7	29.2	4.9	5.7	0.2
Judo	6	19.8 ± 2.6	55.4 ± 5.7	161.2 ± 6.2	21.3 ± 1.7	21.9 ± 4.0	3.2 ± 1.1	4.3 ± 0.8	2.4 ± 1.0
Judo < 44 kg	1	20.0	44.1	145.0	21.0	23.6	5.2	4.5	1.5
Judo < 48 kg	1	20.0	48.7	152.0	21.1	23.5	4.5	4.6	1.9
Judo < 57 kg	2	20.5 ± 0.7	56.9 ± 3.5	160.5 ± 2.1	22.1 ± 2.0	23.0 ± 3.1	3.2 ± 0.5	4.1 ± 0.4	2.0 ± 1.0
Karate	8	18.5 ± 0.9	58.8 ± 11.4	158.9 ± 8.3	23.1 ± 2.6	27.0 ± 4.2	5.0 ± 1.2	4.4 ± 0.8	1.6 ± 0.8
Karate, kata	2	17.5 ± 0.7	51.0 ± 2.9	157.5 ± 5.0	20.6 ± 0.1	21.4 ± 1.4	3.8 ± 0.6	3.2 ± 0.1	2.5 ± 0.4
Karate, kumite	4	18.5 ± 1.0	59.1 ± 4.9	158.2 ± 4.6	23.6 ± 1.2	25.3 ± 1.4	4.3 ± 1.2	4.5 ± 1.0	1.3 ± 0.5
Kickboxing, low kick	3	19.3 ± 1.1	57.8 ± 3.0	163.0 ± 5.3	21.8 ± 0.4	25.7 ± 0.6	4.6 ± 0.5	3.4 ± 0.7	2.3 ± 0.5
Long jump	4	21.4 ± 1.4	62.4 ± 5.7	170.7 ± 7.9	21.4 ± 0.5	23.6 ± 1.3	3.4 ± 0.7	3.1 ± 0.7	2.9 ± 0.6
Olympic wrestling	17	18.9 ± 1.5	57.2 ± 8.5	156.7 ± 6.1	23.2 ± 2.3	23.9 ± 3.8	4.4 ± 1.5	4.8 ± 0.9	1.4 ± 0.7
Olympic wrestling < 53 kg	1	19.0	53.0	157.0	21.5	21.6	3.0	4.6	2.0
Padel, doubles, backhand player	1	21.0	59.2	154.0	25.0	27.7	4.3	5.2	0.7
Padel, doubles, right-handed player	1	23.0	57.2	164.0	21.3	25.5	3.5	3.4	2.6
Pole vault	1	23.0	60.9	164.0	22.6	23.6	2.6	4.8	1.9
Powerlifting < 44 kg	1	26.0	40.3	150.9	17.9	20.7	3.4	2.0	3.6
Powerlifting < 90 kg	1	17.0	88.6	167.0	31.8	37.8	7.5	6.6	0.1
Racewalking, 20 km	1	18.0	48.0	157.0	19.5	22.8	5.5	3.3	3.0
Road cycling	1	17.0	67.1	164.0	24.9	24.6	4.0	4.1	1.1
Rugby	6	19.5 ± 1.4	59.4 ± 9.0	158.5 ± 3.4	23.6 ± 2.8	26.3 ± 4.5	4.8 ± 1.5	4.2 ± 1.1	1.4 ± 1.0
Rugby 7s, prop	1	18.0	73.7	165.2	27.1	26.9	5.5	5.2	0.6
Rugby, center	2	19.0 ± 1.4	54.6 ± 2.9	156.5 ± 6.4	22.4 ± 0.6	24.9 ± 2.3	4.8 ± 1.5	4.0 ± 0.7	1.6 ± 0.6
Rugby, front row	1	27.0	74.5	160.8	29.1	33.6	4.6	6.4	0.1
Rugby, inside center	1	21.0	57.9	161.0	22.3	22.9	3.1	3.6	1.9
Rugby, prop	3	22.0 ± 2.6	66.2 ± 1.1	161.7 ± 2.3	25.3 ± 0.8	28.1 ± 1.5	4.5 ± 1.4	4.8 ± 0.2	0.9 ± 0.2
Rugby, scrum-half	2	19.5 ± 0.7	56.1 ± 3.0	156.8 ± 6.7	23.0 ± 3.1	25.8 ± 8.8	4.6 ± 3.1	4.2 ± 1.5	1.6 ± 1.6
Rugby, wing	7	20.6 ± 1.0	51.7 ± 3.6	158.6 ± 6.3	20.6 ± 1.6	22.9 ± 2.9	3.6 ± 0.9	3.6 ± 1.2	2.6 ± 1.1
Shot put	1	21.0	82.5	164.6	30.3	33.4	4.7	7.7	0.1
Soccer	30	20.8 ± 1.7	56.4 ± 6.3	159.2 ± 6.4	22.3 ± 2.0	23.9 ± 2.8	3.9 ± 1.0	4.3 ± 1.4	1.9 ± 0.9
Soccer, defender	20	19.2 ± 1.3	55.4 ± 7.8	161.3 ± 5.7	21.2 ± 2.4	22.4 ± 3.8	3.9 ± 1.3	3.7 ± 0.9	2.5 ± 1.1
Soccer, forward	13	20.5 ± 2.1	59.0 ± 6.3	161.7 ± 4.2	22.6 ± 2.4	24.5 ± 3.4	4.1 ± 0.8	4.1 ± 1.4	2.0 ± 1.0
Soccer, goalkeeper	7	19.6 ± 1.0	65.1 ± 8.7	162.5 ± 8.1	24.6 ± 1.6	27.9 ± 3.7	5.3 ± 0.8	4.5 ± 0.9	1.2 ± 0.6
Soccer, midfielder	10	20.1 ± 2.4	51.0 ± 7.8	155.7 ± 6.4	20.9 ± 2.3	22.1 ± 4.0	3.4 ± 0.9	4.1 ± 1.4	2.3 ± 1.0
Softball	11	19.8 ± 1.8	60.3 ± 10.9	161.8 ± 7.1	22.9 ± 2.7	26.3 ± 5.6	4.5 ± 1.3	4.0 ± 1.5	1.9 ± 0.8
Softball, fielder	3	18.0 ± 1.0	59.5 ± 5.8	164.0 ± 6.9	22.1 ± 1.8	24.3 ± 4.8	4.6 ± 1.4	3.6 ± 0.7	2.2 ± 1.0
Softball, second base	2	19.5 ± 0.7	53.2 ± 2.0	158.0 ± 0.0	21.3 ± 0.8	21.3 ± 1.1	3.1 ± 0.1	3.7 ± 0.0	2.1 ± 0.3
Softball, shortstop	1	20.0	60.5	163.0	22.8	26.1	4.1	4.5	1.8
Sport climbing	3	22.0 ± 1.0	44.4 ± 3.7	154.0 ± 5.3	18.7 ± 1.7	20.0 ± 1.6	3.3 ± 0.7	2.9 ± 0.6	3.3 ± 1.2
Sprint	7	19.2 ± 0.8	54.9 ± 10.8	162.4 ± 9.6	20.8 ± 2.7	20.5 ± 4.1	2.8 ± 1.0	3.6 ± 1.2	2.9 ± 1.4
Sprint, 100 m	1	21.0	57.7	162.0	22.0	22.7	3.4	3.4	2.1
Sprint, 200 m	3	23.0 ± 2.6	60.5 ± 1.9	164.2 ± 2.6	22.5 ± 0.5	23.6 ± 1.1	3.8 ± 0.4	3.6 ± 0.8	2.0 ± 0.3
Sprint, 400 m	2	23.0 ± 1.4	51.9 ± 4.9	162.0 ± 1.4	19.8 ± 1.5	20.9 ± 1.0	3.0 ± 1.1	2.8 ± 0.7	3.2 ± 0.8
Sprint, 400 m hurdles	1	18.4	63.8	163.7	23.7	21.9	2.6	4.6	1.4
Table tennis	6	20.8 ± 1.4	57.4 ± 7.5	160.5 ± 10.3	22.4 ± 3.8	25.6 ± 4.4	4.7 ± 1.3	4.0 ± 1.9	2.2 ± 1.9
Taekwondo	8	20.0 ± 1.1	60.0 ± 10.2	164.7 ± 5.4	22.0 ± 2.8	24.0 ± 4.7	4.2 ± 1.5	3.8 ± 1.2	2.4 ± 1.3
Taekwondo < 46 kg	1	22.0	48.4	162.0	18.4	18.1	2.4	2.0	4.0
Taekwondo < 49 kg	1	22.0	51.9	165.0	19.1	20.5	2.7	2.6	3.8
Track and field long-distance	4	20.7 ± 1.4	53.5 ± 7.4	158.7 ± 7.2	21.3 ± 2.9	23.6 ± 3.8	4.2 ± 0.9	3.5 ± 0.9	2.4 ± 1.5
Track and field middle-distance	1	23.0	51.3	156.0	21.1	21.9	3.4	4.1	2.2
Track and field, 1500 m	1	20.0	53.9	158.0	21.6	24.0	3.8	2.7	2.0
Track and field, 10,000 m	2	20.5 ± 0.7	50.8 ± 0.0	157.0 ± 5.7	20.6 ± 1.5	24.9 ± 0.8	4.2 ± 0.2	3.6 ± 1.0	2.5 ± 1.1
Track and field, 3000 m steeplechase	1	20.0	47.1	155.0	19.6	18.5	3.1	4.9	2.8
Track and field, 4 × 100 m relay	1	20.0	59.0	162.0	22.5	22.8	3.1	3.9	1.9
Track and field, 5000 m	1	22.0	49.9	156.4	20.5	20.6	2.6	4.8	2.5
Track and field, 800 m	3	19.0 ± 0.0	53.4 ± 4.0	157.1 ± 1.9	21.6 ± 1.1	21.2 ± 2.0	3.0 ± 0.9	4.1 ± 0.2	2.0 ± 0.4
Track cycling	1	21.0	56.9	158.0	22.8	21.9	2.5	5.0	1.5
Triathlon	6	19.2 ± 1.3	58.1 ± 4.7	160.7 ± 7.2	22.5 ± 1.4	24.8 ± 2.7	4.4 ± 1.0	4.1 ± 0.6	1.8 ± 0.9
Volleyball	21	20.7 ± 1.8	70.0 ± 11.0	172.4 ± 7.8	23.5 ± 2.6	26.0 ± 3.7	4.3 ± 1.3	3.6 ± 1.1	2.2 ± 1.1
Volleyball, center	1	21.0	76.2	183.0	22.8	27.3	5.9	3.1	3.0
Volleyball, outside hitter	1	21.0	70.4	166.0	25.5	29.5	6.0	6.4	1.0
Volleyball, setter	1	19.0	66.1	167.0	23.7	24.5	3.3	4.9	1.7
Weightlifting	4	20.8 ± 2.6	55.6 ± 8.4	154.8 ± 4.1	23.2 ± 3.5	24.0 ± 4.0	3.7 ± 0.9	4.8 ± 1.6	1.4 ± 1.0
Weightlifting < 45 kg	1	22.0	47.6	155.0	19.8	18.7	2.1	4.5	2.7
Weightlifting < 55 kg	1	21.0	55.8	154.0	23.5	23.6	3.8	5.4	1.0
Weightlifting < 59 kg	2	18.5 ± 0.7	62.8 ± 2.6	152.0 ± 0.0	27.2 ± 1.1	28.6 ± 3.0	5.2 ± 1.1	6.8 ± 0.1	0.2 ± 0.1
Weightlifting < 64 kg	1	24.0	66.1	153.0	28.2	29.3	5.7	6.1	0.1
TOTAL	412								

Note. This table shows the sample size (*n*), means of somatotype components (endomorphy, mesomorphy, and ectomorphy) according to the Heath–Carter method, body mass (kg), height (cm), body mass index (BMI, kg/m^2^), and body fat percentage estimated using Equation 5 proposed by Lean et al. [45].

**Table 3:** Descriptive table of body composition and somatotype characteristics in male athletes by sport macro-category.

Sport Macro-Category	Combat Sports (*n* = 87)	Endurance Events (*n* = 13)	Individual Sports (*n* = 44)	Sprint Events (*n* = 21)	Team Sports (*n* = 310)	Track and Field (*n* = 2)	*p* Value
Age	20.0 ^a^ (18.2–22.0)	20.0 (19.0–22.0)	20.0 (19.0–21.0)	22.0 (19.3–23.0)	21.0 ^b^ (20.0–22.5)	19.0 (18.0–20.0)	<0.001 **
Body mass (kg)	68.5 ^a^ (62.5–75.6)	63.9 ^a^ (59.4–74.4)	72.3 ^a^ (65.9–79.0)	73.6 (64.5–79.4)	79.6 ^b^ (71.2–88.9)	79.2 (73.0–85.4)	<0.001 **
Height (cm)	170.0 ^a,b,c^ (167.0–175.0)	170.0 ^a,b,e^ (168.0–174.0)	173.2 ^a,b,c,e^ (168.5–178.7)	176.2 ^b,c,d,e^ (174.0–185.4)	178.0 ^d,e^ (172.0–183.0)	175.0 ^a,c,d,e^ (175.0–175.0)	<0.001 **
BMI (kg/m^2^)	23.5 ^a^ (21.7–25.9)	22.5 ^a^ (21.0–23.8)	23.3 (22.2–26.1)	22.4 ^a^ (20.5–24.2)	25.2 ^b^ (23.1–27.4)	25.8 (23.8–27.9)	<0.001 **
%BF (Lean et al.) [45]	11.4 ^a^ (8.8–14.6)	9.7 (7.3–17.5)	10.5 ^a^ (7.6–16.7)	6.9 ^b^ (5.6–8.4)	12.5 ^a^ (9.3–16.1)	11.6 (10.4–12.9)	<0.001 **
Endomorphy	2.8 ^a^ (2.1–3.6)	2.0 (1.8–3.7)	2.7 ^a^ (1.9–3.9)	1.6 ^b^ (1.1–2.0)	2.9 ^a^ (2.2–3.8)	2.5 (2.3–2.7)	<0.001 **
Mesomorphy	5.4 (4.5–6.2)	4.3 (3.6–5.4)	5.0 (4.0–6.1)	4.5 ^a^ (3.9–5.1)	5.5 ^b^ (4.7–6.5)	6.2 (5.7–6.7)	<0.001 **
Ectomorphy	1.9 ^a^ (1.1–2.8)	2.2 (1.9–3.1)	2.1 (1.3–3.0)	3.0 ^b^ (2.2–4.0)	1.7 ^a^ (1.0–2.5)	1.4 (0.8–2.1)	<0.001 **

Note. Data are shown as median and interquartile range (Q1–Q3). Different superscript letters (^a,b,c,d,e^) within the same row indicate statistically significant differences between sport macro-categories according to Dunn’s post hoc test with Bonferroni correction after a Kruskal–Wallis analysis (*p* < 0.05). %BF = body fat percentage; BMI = body mass index. ** *p* < 0.001.

**Table 4:** Descriptive table of body composition and somatotype characteristics in female athletes by sports macro-category.

Sport Macro-category	Combat Sports (*n* = 65)	Endurance Events (*n* = 22)	Individual Sports (*n* = 41)	Sprint Events (*n* = 15)	Team Sports (*n* = 265)	Track and Field (*n* = 4)	*p* Value
Age	19.0 ^b^ (18.0–20.2)	20.0 (19.0–21.0)	21.0 ^a^ (19.0–22.2)	20.0 (19.0–21.7)	20.0 ^a^ (19.0–21.5)	21.0 (20.0–21.5)	0.011 *
Body mass (kg)	56.1 ^a,b,c,d,e^ (51.7–62.2)	51.5 ^a,b,c,d^ (49.2–57.1)	58.1 ^a,b,c,d,e^ (52.8–60.9)	57.7 ^a,b,c,d,e^ (49.2–60.1)	59.3 ^a,c,d,e^ (54.1–66.0)	81.2 (78.1–89.6)	<0.001 **
Height (cm)	160.0 (154.8–164.2)	157.2 ^a^ (155.3–159.0)	156.5 ^a^ (154.0–164.0)	162.8 (161.2–164.2)	161.6 ^b^ (158.0–167.0)	164.8 (164.0–165.0)	<0.001 **
BMI (kg/m^2^)	22.2 ^a,b,c,d,e^ (20.9–23.7)	21.0 ^a,b,c,d^ (20.2–22.2)	22.2 ^a,b,c,d,e^ (20.2–24.2)	22.0 ^a,b,c,d,e^ (19.4–22.8)	22.8 ^a,c,d,e^ (21.1–24.3)	29.8 (29.0–32.9)	<0.001 **
%BF (Lean et al.) [45]	24.8 ^a,b,c,d,e^ (21.9–27.0)	22.9 ^a,b,c,d,e^ (20.6–24.4)	23.5 ^a,b,c,d,e^ (21.1–25.4)	21.9 ^a,b,c,d^ (20.2–23.1)	25.0 ^a,b,c,e,f^ (22.4–27.4)	31.9 ^e,f^ (29.8–38.2)	<0.001 **
Endomorphy	4.3 ^a^ (3.2–5.2)	3.8 (3.1–4.5)	3.7 (2.7–4.5)	3.2 ^b^ (2.5–3.7)	4.2 ^a^ (3.3–5.0)	5.0 ^a^ (4.8–7.0)	<0.001 **
Mesomorphy	4.2 ^a^ (3.5–4.9)	4.1 ^a^ (3.3–4.4)	4.8 (3.2–5.4)	3.6 ^a^ (2.6–4.2)	4.1 ^a^ (3.3–4.9)	7.0 ^b^ (6.0–8.1)	0.002 **
Ectomorphy	1.8 ^a^ (1.2–2.3)	2.3 ^a^ (1.6–2.9)	1.7 ^a^ (0.9–2.7)	2.2 ^a^ (1.6–3.3)	1.8 ^a^ (1.1–2.6)	0.1 ^b^ (0.1–0.1)	0.002 **

Note. Data are shown as median and interquartile range (Q1–Q3). Different superscript letters (^a,b,c,d,e^) within the same row indicate statistically significant differences between sport macro-categories according to Dunn’s post hoc test with Bonferroni correction after a Kruskal–Wallis analysis (*p* < 0.05). %BF = body fat percentage; BMI = body mass index. * *p* < 0.05; ** *p* < 0.001.

## Data Availability

The original contributions and data created in this study are included in the article/supplementary materials. Further inquiries can be directed to the corresponding author (pep.tur@uib.es).

## Supplementary Materials

The following supporting information can be downloaded at: https://www.mdpi.com/article/10.3390/jfmk10030329/s1, Figure S1: Somatocharts from different sports disciplines in male athletes; Figure S2: Somatocharts from different sports disciplines in male athletes; Table S1: Descriptive characteristics across Mexican male athletes; Table S2: Descriptive characteristics of Mexican female athletes.

## Abbreviations

The following abbreviations are used in this manuscript:

STROBE	Strengthening the Reporting of Observational Studies in Epidemiology
UANL	Universidad Autónoma de Nuevo León
CONADE	Comisión Nacional de Cultura Física y Deporte
CONADEIP	Comisión Nacional Deportiva Estudiantil de Instituciones Privadas
ONEFA	Organización Nacional Estudiantil de Futbol Americano
TEM	Technical Error of Measurement
ISAK	International Society for the Advancement of Kinanthropometry
BMI	Body Mass Index
%BF	Body Fat Percentage

## Appendix A

### Appendix A.1

The endomorphy component was obtained using the following equation:

$$Endomorphy=-0.7182+0.1451\times \sum SS-0.00068\times ({\sum SS)}^{2}+0.0000014\times ({\sum SS)}^{3}$$

where:

∑SS=(skinfolds:triceps+subscapular+supraspinale)×(170.18/height (cm))

The mesomorphy component was calculated using the following equation:

$$\begin{array}{ll} Mesomorphy= & \left(0.858\times humerus\;breadth)+(0.601\times femur\;breadth\right)\\ & +(0.188\times corrected\;flexed\;arm\;girth)\\ & +(0.161\times corrected\;calf\;girth)-(0.131\times height(cm))+4.5 \end{array}$$

The ectomorphy component was calculated based on the height–weight ratio (HWR), defined as height divided by the cube root of body mass. The classification of ectomorphy was established according to the following criteria:

If HWR≥40.75, Ectomorphy=(0.732×HWR)−28.58

If HWR>38.25 and<40.75,      Ectomorphy=(0.463×HWR)−17.63

If HWR≤38.25, Ectomorphy=0.1

where:

$$HWR=\frac{height\;(cm)}{∛weight\;(kg)}$$

### Appendix A.2

The X and Y axes are used for the somatochart, calculated using the following equations:

X=ectomorphy component−endomorphy component

Y=2×mesomorphy component−(endomorphy component+ectomorphy component)
