# Supplementary material for: A National Study of Somatotypes in Mexican Athletes Across 43 Sports
Source: J Funct Morphol Kinesiol. 2025 Aug 27;10(3):329. doi: 10.3390/jfmk10030329 (PMC12452521; doi:10.3390/jfmk10030329)

Figure S1. Somatocharts from different sports disciplines in male athletes.

Figure S1a. Somatochart in team sports

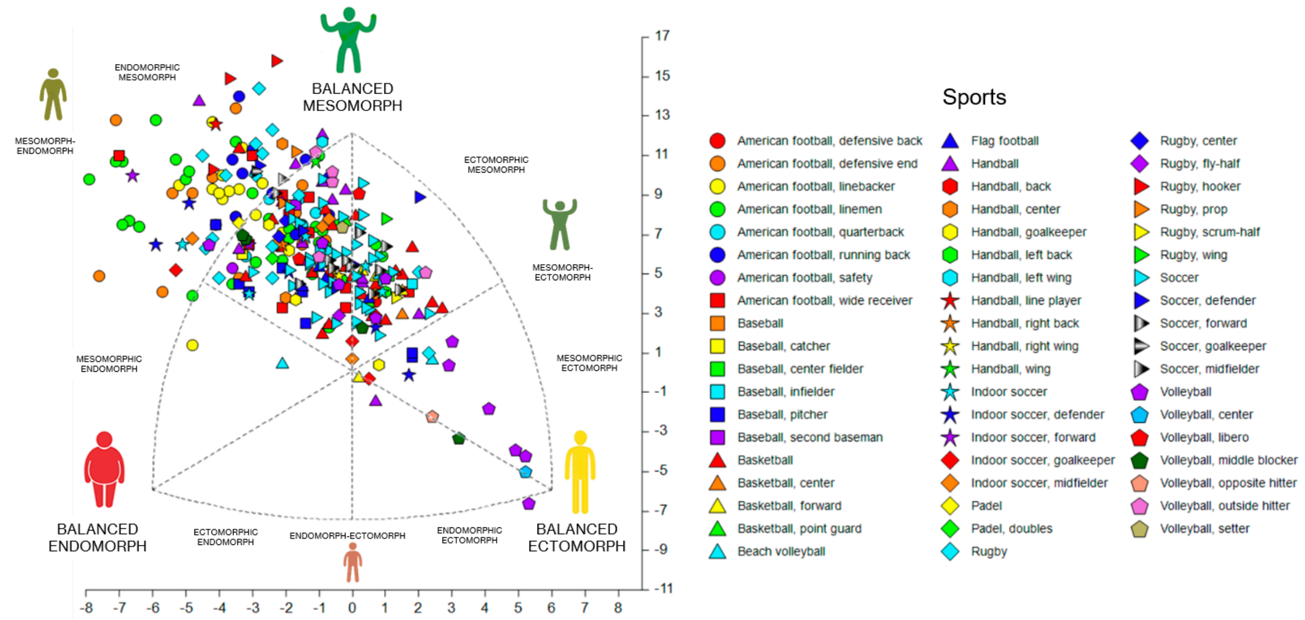

Figure S1b. Somatochart in combat sports

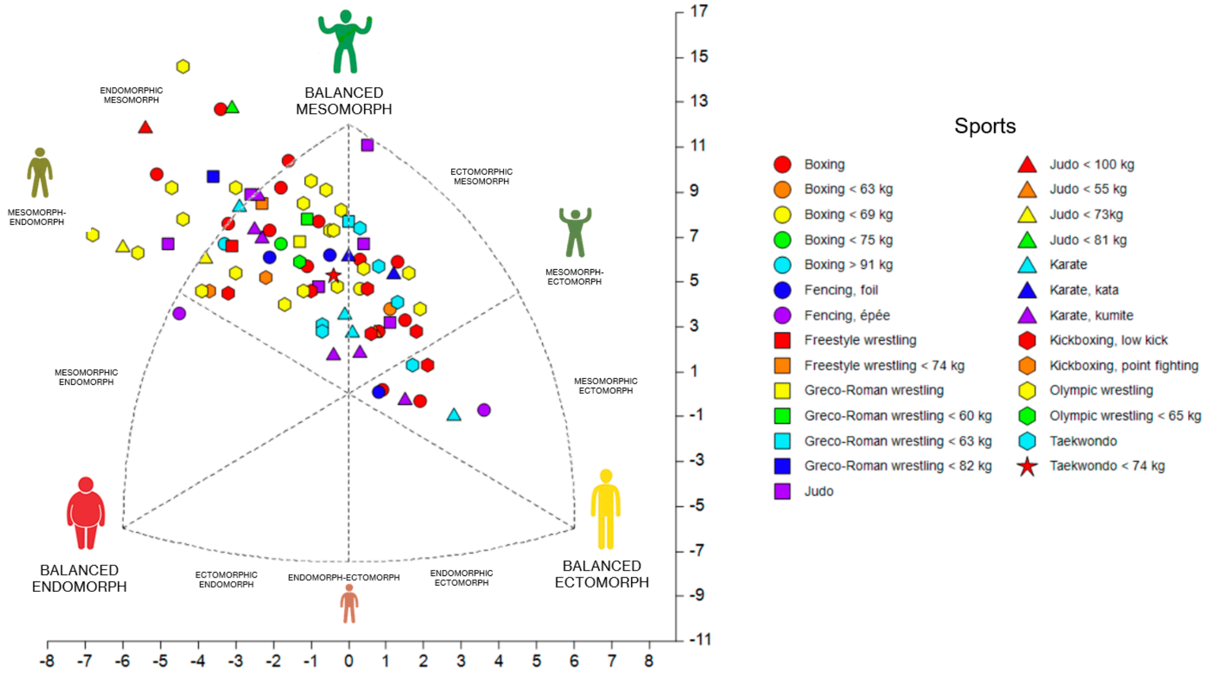

Figure S1c. Somatochart in individual sports.

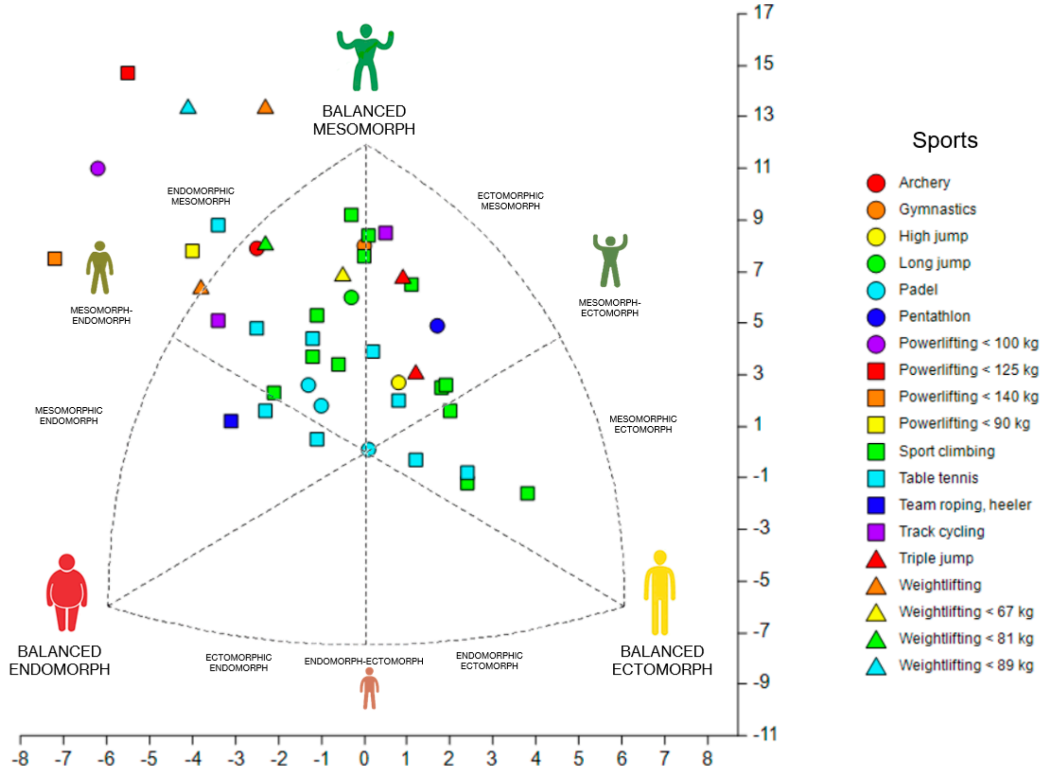

Figure S1d. Somatochart on endurance events

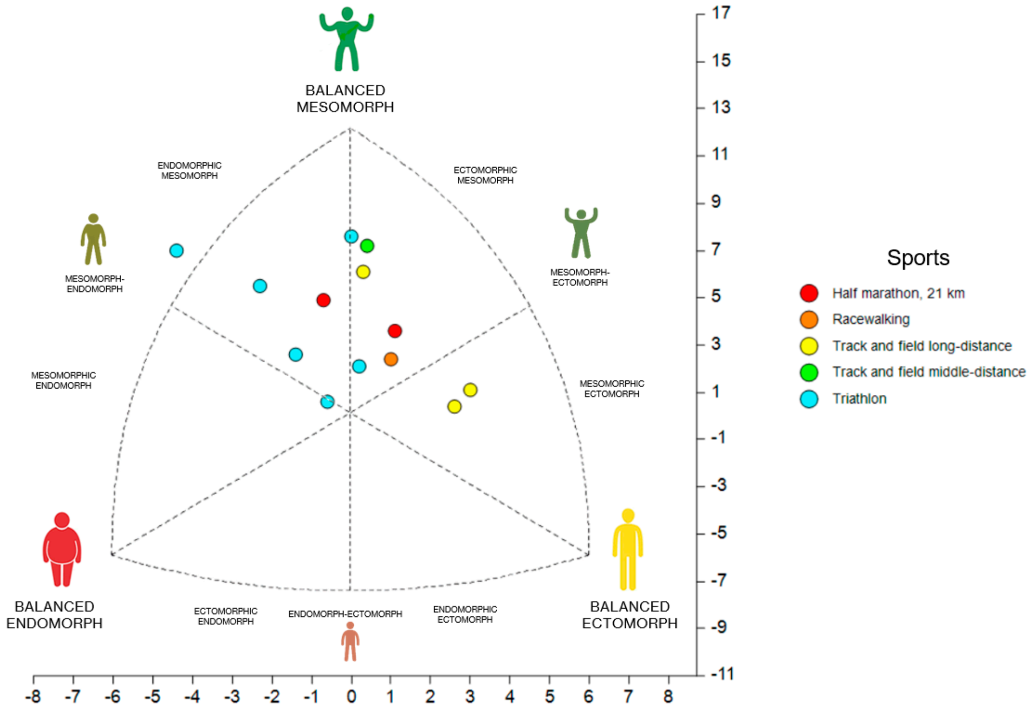

Figure S1e. Somatochart in sprint events

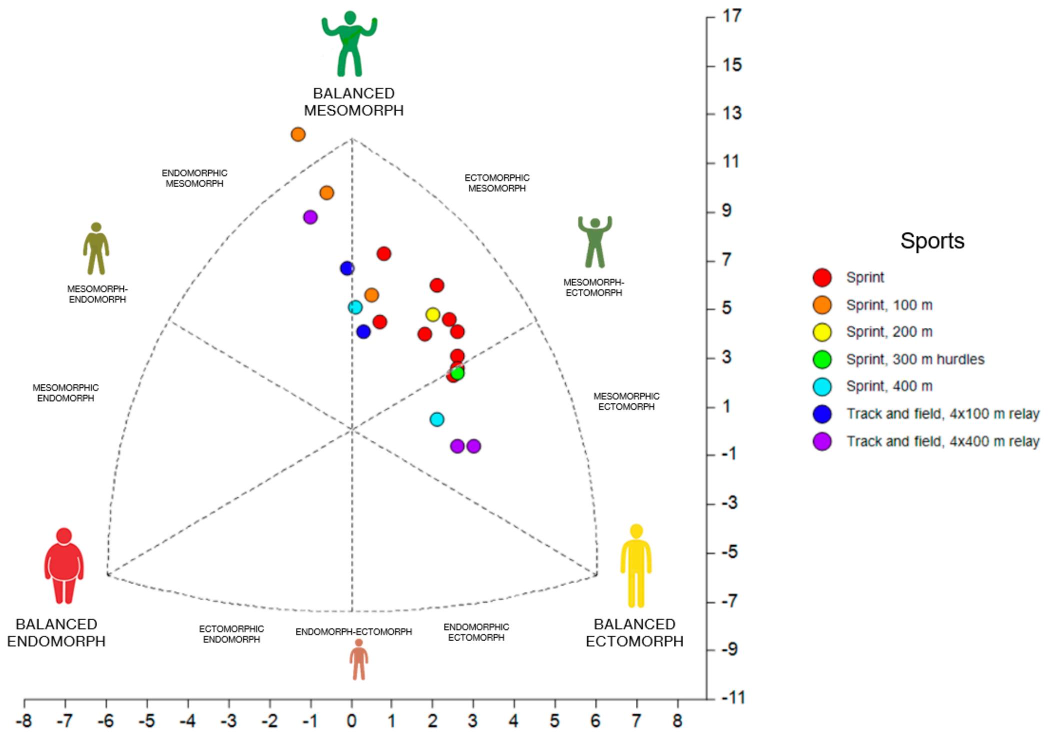

Figure S1f. Somatochart on throwing events

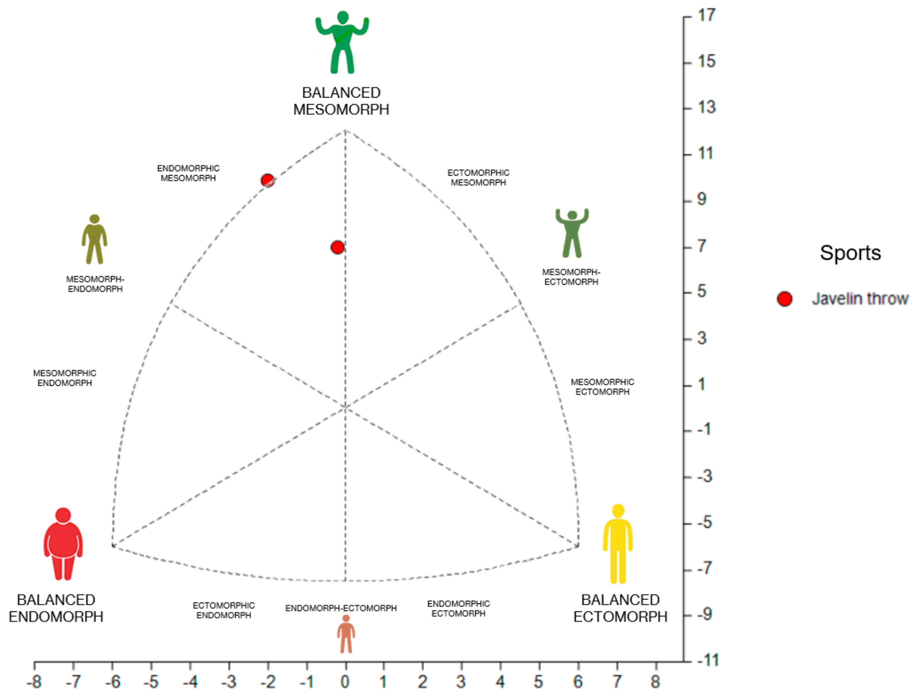

Supplement: Supplementary file 1 [file jfmk-10-00329-s001.zip › Supplementary Material Figure S1 somatocharts male athletes.pdf]
