# Supplementary material for: A National Study of Somatotypes in Mexican Athletes Across 43 Sports
Source: J Funct Morphol Kinesiol. 2025 Aug 27;10(3):329. doi: 10.3390/jfmk10030329 (PMC12452521; doi:10.3390/jfmk10030329)

Figure S2. Somatocharts from different sports disciplines in male athletes.

Figure S2a. Somatochart in team sports

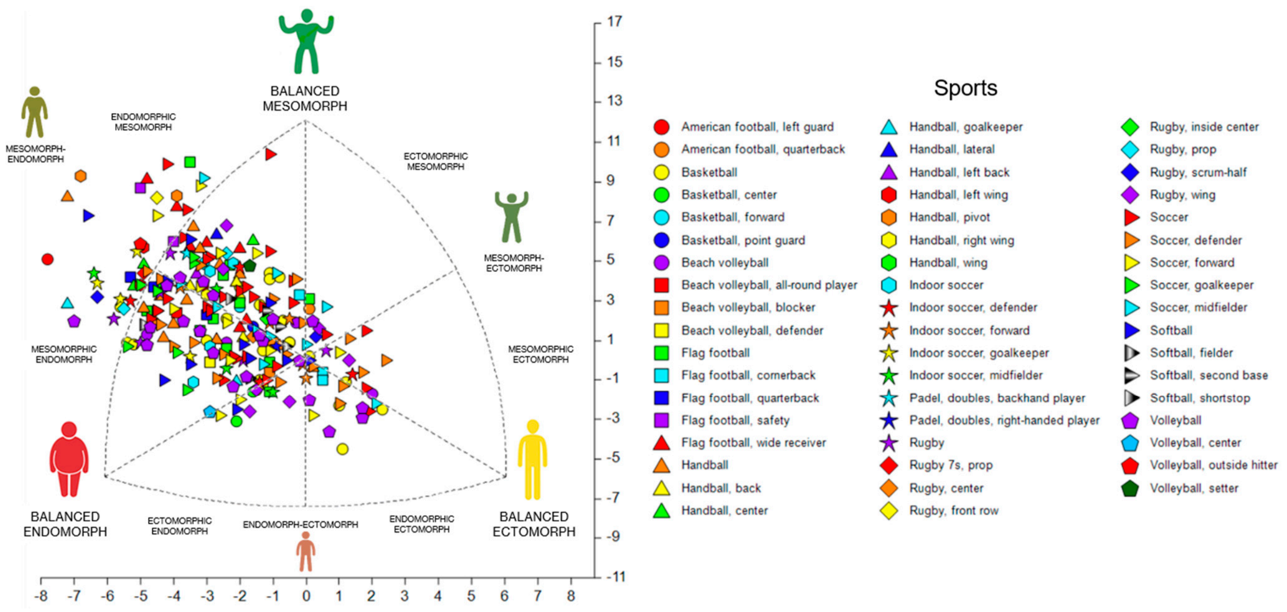

Figure S2b. Somatochart in combat sports

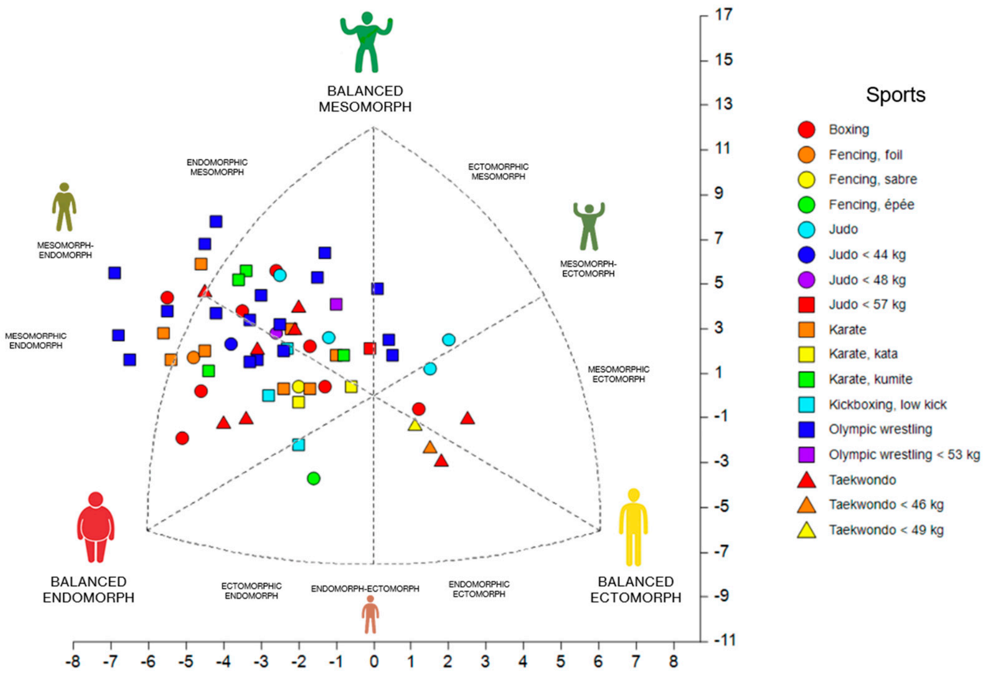

Figure S2c. Somatochart in individual sports.

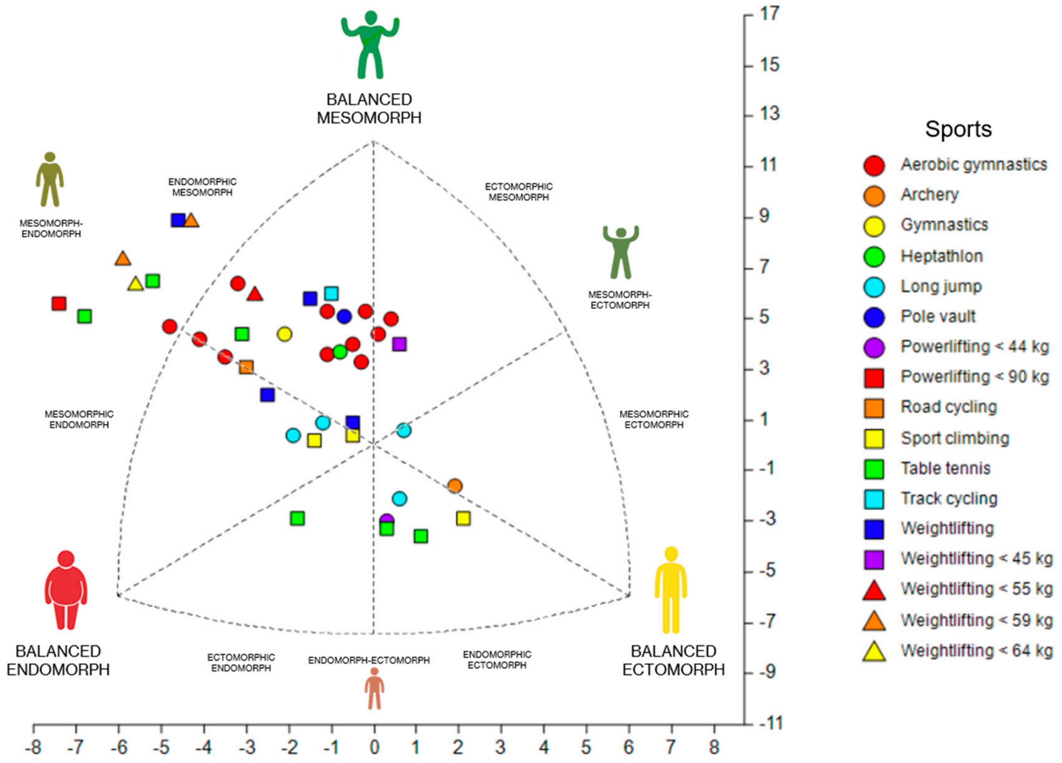

Figure S2d. Somatochart on endurance events

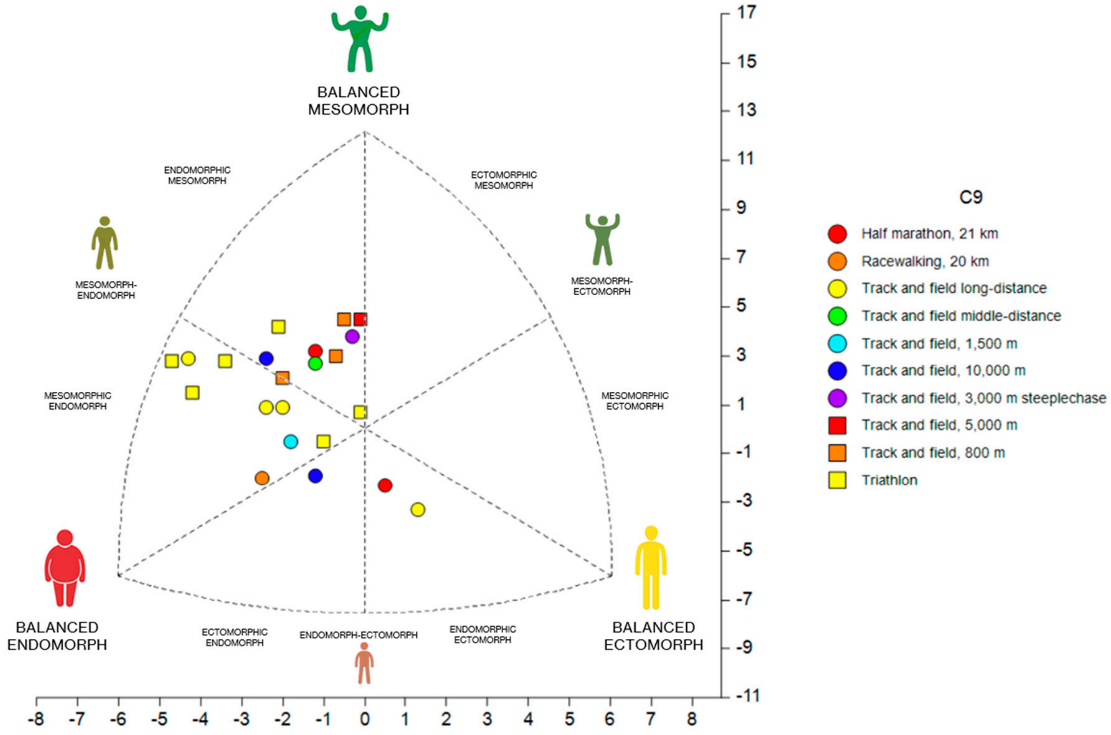

Figure S2e. Somatochart on sprint events

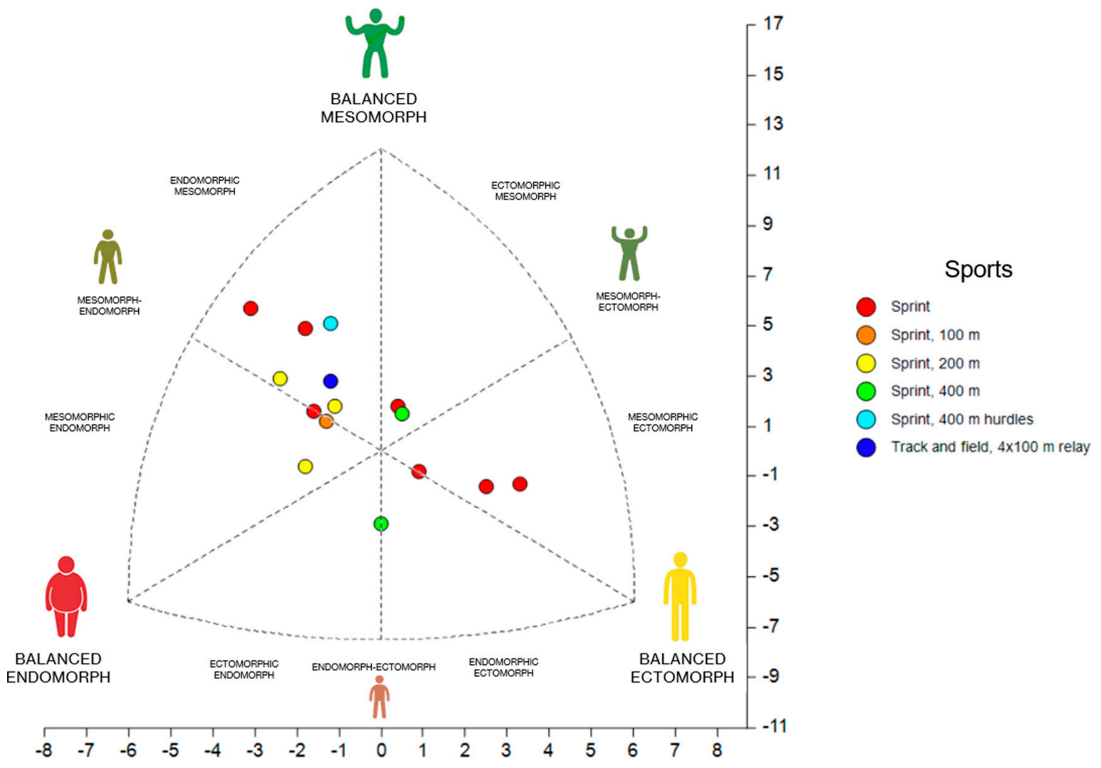

Figure S2f. Somatochart in track and field

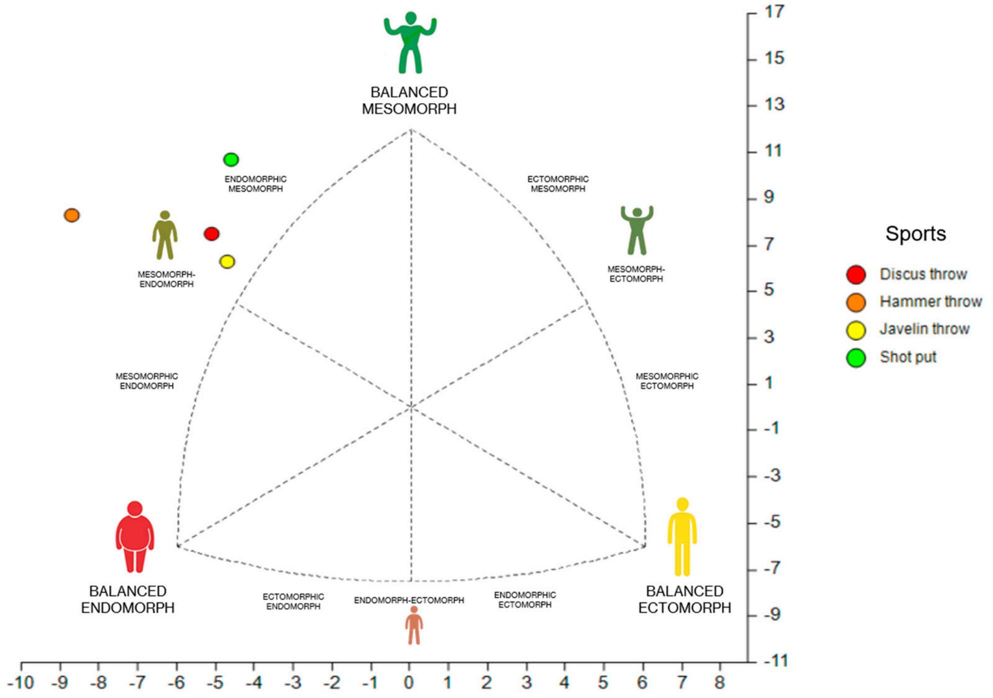

Supplement: Supplementary file 1 [file jfmk-10-00329-s001.zip › Supplementary Material Figure S2 somatocharts female athletes.pdf]
