# Supplementary material for: A National Study of Somatotypes in Mexican Athletes Across 43 Sports
Source: J Funct Morphol Kinesiol. 2025 Aug 27;10(3):329. doi: 10.3390/jfmk10030329 (PMC12452521; doi:10.3390/jfmk10030329)
Supplement: Supplementary file 1 [file jfmk-10-00329-s001.zip › Supplementary material Table S1 male mexican athletes.pdf]

Table S1. Descriptive characteristics across Mexican male athletes.

SPORT	*n*	BODY MASS (kg)	HEIGHT (cm)	BMI (kg/m²)	%BF	Somatotype rating
American football, defensive back	1	76.4	182.0	23.1	9.9	2.2-4.3-2.8
American football, defensive end	14	110.4	184.8	32.4	21.6	4.9-7.0-0.7
American football, linemen	31	94.3	178.6	29.4	16.6	4.1-6.4-1.1
American football, lineback	15	93.7	178.9	29.3	15.7	4.2-6.8-0.6
American football, quarterback	5	93.6	184.8	27.4	15.0	3.3-6.2-1.3
American football, running back	11	80.6	171.2	27.5	12.2	3.2-7.0-0.8
American football, safety	1	87.4	177.0	27.9	17.1	4.4-5.3-0.8
American football, wide receiver	11	85.0	178.7	26.6	13.7	3.2-5.7-1.6
Archery	1	89.1	180.0	27.5	14.8	3.5-6.2-1.0
Baseball	1	71.5	170.0	24.7	15.2	3.2-6.6-1.4
Baseball, catcher	1	92.7	182.0	28.0	17.2	4.3-5.6-1.0
Baseball, center fielder	1	59.7	168.0	21.2	7.3	1.8-4.8-2.9
Baseball, infielder	1	64.8	175.0	21.2	6.7	1.5-4.6-3.3
Baseball, pitcher	8	77.9	177.9	24.6	15.9	3.5-4.6-2.0
Baseball, second baseman	1	76.5	173.0	25.6	13.4	2.9-6.4-1.3
Basketball	25	87.0	186.5	25.0	11.5	2.6-5.1-2.3
Basketball, center	2	83.9	190.0	23.2	7.5	1.8-4.3-3.2
Basketball, forward	4	79.2	185.0	23.2	9.4	2.3-4.4-3.0
Basketball, point guard	3	74.1	177.0	23.7	9.5	2.1-4.9-2.3
Beach volleyball	3	77.5	184.0	22.8	11.7	2.7-4.0-3.1
Boxing	14	70.4	171.4	23.9	12.2	2.9-5.6-2.0
Boxing < 63 kg	1	64.3	174.0	21.2	11.7	2.1-4.6-3.2
Boxing < 69 kg	1	70.2	174.0	23.2	9.4	2.0-4.5-2.3
Boxing < 75 kg	1	76.9	175.0	25.1	14.7	3.3-5.8-1.5
Boxing > 91 kg	1	94.3	184.0	27.9	15.5	4.4-6.1-1.1
Fencing, épée	2	73.4	178.0	22.6	15.6	3.6-4.1-3.1
Fencing, foil	3	62.0	167.7	22.1	12.6	3.1-4.8-2.5
Flag football	4	81.8	182.0	24.7	13.6	2.8-4.8-2.1

Freestyle wrestling	1	91.8	182.0	27.7	13.1	4.1-5.9-1.0
Freestyle wrestling < 74 kg	1	75.7	168.0	26.8	12.3	3.0-6.2-0.8
Greco-Roman wrestling	1	68.1	167.0	24.4	9.7	2.7-5.4-1.4
Greco-Roman wrestling < 60 kg	1	63.2	162.0	24.1	10.1	2.3-5.7-1.2
Greco-Roman wrestling < 63 kg	1	64.7	167.0	23.2	7.6	1.9-5.7-1.9
Greco-Roman wrestling < 82 kg	1	83.7	169.0	29.3	14.1	3.9-6.9-0.3
Gymnastics	1	62.5	166.0	22.7	7.3	2.1-6.1-2.0
Half marathon, 21 km	2	72.7	177.0	23.1	9.2	2.3-4.6-2.5
Handball	14	76.9	175.5	24.9	10.7	2.9-6.0-1.8
Handball, back	1	93.2	184.0	27.5	17.0	3.1-6.2-1.2
Handball, center	3	70.8	170.3	24.3	13.0	2.9-5.5-1.7
Handball, goalkeeper	4	81.4	180.8	24.9	16.3	3.4-5.0-2.1
Handball, left back	1	78.6	186.0	22.7	9.3	2.2-4.7-3.2
Handball, left wing	1	70.2	164.0	26.1	6.8	1.7-7.1-0.8
Handball, line player	1	93.5	174.0	30.9	16.6	4.3-8.5-0.1
Handball, right back	2	88.0	182.5	26.6	16.8	3.3-5.9-1.5
Handball, right wing	2	69.8	173.5	23.2	11.5	2.7-5.1-2.3
Handball, wing	1	71.2	165.0	26.2	8.4	1.9-6.7-0.8
High jump	1	70.0	178.4	22.1	12.1	2.3-4.1-3.1
Indoor soccer	5	71.3	170.1	24.3	14.0	3.5-5.3-1.8
Indoor soccer, defender	5	79.8	176.0	25.8	15.5	3.8-5.0-1.7
Indoor soccer, forward	2	77.5	164.0	28.9	16.7	5.3-7.0-0.5
Indoor soccer, goalkeeper	4	69.7	174.3	22.9	16.1	3.6-4.2-2.5
Indoor soccer, midfielder	5	70.2	170.4	24.2	14.8	3.5-5.6-1.8
Javelin throw	2	79.2	175.0	25.9	11.7	2.5-6.2-1.4
Judo	7	65.2	167.0	23.4	11.6	2.9-5.7-1.9
Judo < 100 kg	1	97.2	170.0	33.6	17.3	5.5-8.8-0.1
Judo < 55 kg	1	53.5	164.0	19.9	11.2	2.6-4.3-3.3
Judo < 73kg	2	82.0	167.5	27.7	19.8	5.4-6.2-0.5
Judo < 81 kg	1	81.2	164.0	30.2	10.3	3.2-8.1-0.1
Karate	4	69.5	174.8	22.7	12.2	2.8-4.5-2.8

Karate, kata	2	56.4	162.5	21.3	7.2	1.9-5.1-2.5
Karate, kumite	6	72.3	174.8	23.7	14.1	3.2-4.9-2.2
Kickboxing, low kick	6	64.6	171.2	22.0	10.9	2.6-4.4-2.7
Kickboxing, point fighting	2	76.6	175.0	24.9	15.9	4.6-5.6-1.6
Long jump	1	70.1	173.0	23.4	10.4	2.5-5.3-2.1
Olympic wrestling	20	73.7	170.1	25.4	12.4	3.4-6.0-1.5
Olympic wrestling < 65 kg	1	66.3	168.0	23.5	9.9	3.1-5.4-1.8
Padel	4	74.3	175.0	24.2	15.3	3.5-4.3-2.1
Padel, doubles	1	77.6	176.0	25.1	18.1	4.0-5.7-1.6
Pentathlon	1	76.2	184.5	22.5	6.8	1.6-4.9-3.3
Powerlifting < 90 kg	1	80.1	170.0	27.7	17.8	4.7-6.5-0.6
Powerlifting < 125 kg	1	128.4	179.0	40.1	34.7	5.6-10.2-0.1
Powerlifting < 100 kg	1	100.3	166.0	36.4	26.4	6.3-8.7-0.1
Powerlifting < 140 kg	1	131.6	190.0	36.5	27.7	7.3-7.5-0.1
Racewalking	1	59.5	168.0	21.1	7.4	2.0-3.6-2.9
Rugby	16	78.6	172.6	26.5	14.5	3.6-6.5-1.2
Rugby, center	2	75.0	174.0	25.6	16.8	3.4-6.0-1.3
Rugby, fly-half	1	63.1	170.0	21.8	12.1	3.0-4.3-2.7
Rugby, hooker	3	86.9	168.3	29.8	12.6	3.6-8.7-0.2
Rugby, prop	2	83.8	176.3	27.0	13.2	2.9-6.9-1.0
Rugby, scrum-half	1	74.0	180.0	22.8	5.3	1.5-4.0-2.8
Rugby, wing	1	66.2	171.0	22.6	5.1	1.4-5.8-2.4
Soccer	43	73.7	175.9	23.8	11.6	2.6-5.2-2.2
Soccer, defender	6	72.9	173.7	24.2	11.0	2.5-5.6-2.0
Soccer, forward	7	68.9	170.3	23.3	9.1	2.5-5.3-2.1
Soccer, goalkeeper	1	81.7	173.0	27.3	12.7	3.7-7.4-0.8
Soccer, midfielder	6	58.0	171.9	22.9	10.4	2.4-5.3-2.3
Sport climbing	13	62.9	170.8	21.5	9.6	2.3-4.6-2.9
Sprint	9	70.6	179.5	21.8	5.6	1.3-4.4-3.3
Sprint, 100 m	3	76.0	173.3	25.2	8.1	2.0-6.3-1.5
Sprint, 200 m	1	73.5	181.0	22.4	5.6	1.1-4.5-3.0

Sprint, 300 m hurdles	1	59.7	174.0	19.7	6.7	1.4-3.9-4.0
Sprint, 400 m	2	77.3	186.0	22.4	8.8	2.3-4.3-3.4
Table tennis	9	69.5	173.8	23.1	12.0	3.1-4.2-2.5
Taekwondo	6	67.4	174.2	22.1	9.6	2.4-4.6-2.8
Taekwondo < 74 kg	1	71.9	174.0	23.7	14.0	2.5-4.9-2.1
Team roping, heeler	1	82.4	184.5	24.3	26.1	5.5-4.6-2.5
Track and field, 4x100 m relay	2	72.8	175.0	23.7	9.0	2.0-4.8-2.1
Track and field, 4x400 m relay	3	74.0	182.7	22.3	8.5	1.8-3.9-3.4
Track and field long-distance	3	58.9	171.1	20.2	7.8	1.6-3.9-3.6
Track and field middle-distance	1	59.1	164.0	22.0	7.3	1.8-5.6-2.2
Track cycling	2	80.2	175.1	26.1	11.2	2.7-5.4-1.2
Triathlon	6	69.5	171.7	23.6	15.6	3.5-4.9-2.1
Triple jump	2	79.0	183.9	23.3	10.6	1.7-4.7-2.8
Volleyball	12	75.7	186.3	22.0	9.7	2.3-3.8-3.8
Volleyball, center	1	73.5	200.0	18.4	6.1	1.2-1.3-6.4
Volleyball, libero	2	69.2	169.5	24.0	8.9	2.3-5.7-1.7
Volleyball, middle blocker	4	91.6	190.8	25.2	14.6	3.4-4.6-2.6
Volleyball, opposite hitter	1	85.9	200.0	21.5	12.9	2.2-2.3-4.6
Volleyball, outside hitter	5	81.5	179.7	25.2	10.7	2.1-6.2-1.9
Volleyball, setter	1	71.3	171.0	24.4	5.2	1.9-5.5-1.6
Weightlifting	2	83.7	171.5	28.4	13.1	3.6-7.0-0.5
Weightlifting < 67 kg	1	68.3	169.0	23.9	6.6	2.1-5.4-1.7
Weightlifting < 81 kg	1	80.8	175.0	26.4	12.2	3.4-6.3-1.1
Weightlifting < 89 kg	1	89.4	167.0	32.1	9.9	4.2-8.9-0.1
TOTAL	480					

Note. The sample size (n), the mean values of the somatotype components (endomorphy, mesomorphy, and ectomorphy) according to the Heath-Carter method, body mass (kg), stature (cm), body mass index (BMI, kg/m^2), and body fat percentage estimated using the equation proposed by Lean et al. (1996) are presented.
