# Supplementary material for: A National Study of Somatotypes in Mexican Athletes Across 43 Sports
Source: J Funct Morphol Kinesiol. 2025 Aug 27;10(3):329. doi: 10.3390/jfmk10030329 (PMC12452521; doi:10.3390/jfmk10030329)
Supplement: Supplementary file 1 [file jfmk-10-00329-s001.zip › Supplementary material Table S2 female mexican athletes.pdf]

Table S2. Descriptive characteristics of Mexican female athletes.

SPORT	*n*	AGE	BODY MASS (kg)	HEIGHT (cm)	BMI (kg/m²)	%BF	Somatotype rating
Aerobic gymnastics	11	20.2	56.4	158.7	22.5	23.0	3.5-4.9-1.8
American football, left guard	1	22.0	89.2	168.1	31.6	37.9	7.9-6.6-0.1
American football, quarterback	1	24.0	62.4	169.6	21.8	23.1	2.6-4.0-2.7
Archery	1	17.0	44.0	159.0	17.4	16.1	2.5-2.7-4.4
Basketball	22	20.4	69.0	172.6	22.8	24.4	3.8-3.6-2.4
Basketball, center	2	22.0	78.9	183.0	23.5	27.7	4.5-2.4-2.7
Basketball, forward	1	25.0	66.3	167.0	23.8	27.0	3.6-4.0-1.6
Basketball, point guard	1	20.0	56.2	160.0	22.0	22.8	3.6-3.6-2.0
Beach volleyball	4	20.5	61.3	166.8	22.1	22.8	3.8-3.4-2.5
Beach volleyball, all-round player	1	22.0	67.8	162.0	25.8	27.1	4.6-3.9-0.8
Beach volleyball, blocker	2	21.0	67.3	173.0	22.5	24.2	3.6-3.1-2.6
Beach volleyball, defender	3	21.3	58.5	163.3	21.9	23.4	4.6-3.2-2.2
Boxing	8	20.8	59.7	161.5	22.9	25.9	4.7-4.1-1.8
Discus throw	1	21.0	80.0	165.0	29.4	30.5	5.2-6.4-0.1
Fencing, épée	1	18.0	67.9	178.0	21.4	25.8	5.0-2.3-3.4
Fencing, foil	1	22.0	63.1	163.3	23.7	31.1	6.2-4.7-1.4
Fencing, sabre	1	19.0	55.5	161.0	21.4	24.3	4.3-3.5-2.3
Flag football	11	20.0	58.1	160.2	22.7	24.7	4.1-4.3-1.8
Flag football, cornerback	3	17.3	50.3	159.7	19.8	19.4	2.8-3.2-3.1
Flag football, quarterback	3	23.0	67.6	164.7	24.7	30.3	5.6-5.1-1.3
Flag football, safety	2	22.5	70.9	162.3	27.0	29.8	5.1-6.5-0.5
Flag football, wide receiver	10	19.7	59.2	157.9	23.8	25.8	4.3-5.1-1.3
Gymnastics	1	21.0	60.4	162.0	23.0	23.5	3.7-4.9-1.6
Half marathon, 21 km	2	20.5	49.3	158.0	19.9	21.4	3.3-3.4-3.0
Hammer throw	1	19.0	96.7	165.0	35.5	43.1	8.8-8.6-0.1
Handball	13	20.6	64.2	163.2	24.2	26.7	4.7-4.8-1.4
Handball, back	3	20.3	60.9	162.0	23.1	24.4	4.1-4.4-1.6
Handball, center	3	20.3	56.7	153.3	24.2	27.2	4.8-5.3-0.9

Handball, goalkeeper	2	21.5	66.6	164.5	24.7	29.8	6.1-4.3-1.5
Handball, lateral	1	19.0	69.2	165.0	25.4	26.0	3.7-5.5-1.0
Handball, left back	1	20.0	70.0	165.0	25.7	28.3	4.2-4.7-0.9
Handball, left wing	3	20.3	63.8	158.3	25.4	26.7	5.0-5.1-0.7
Handball, pivot	2	20.5	73.0	161.0	28.1	32.4	5.7-7.4-0.4
Handball, right wing	1	20.0	58.8	160.0	23.0	25.7	4.5-4.7-1.5
Handball, wing	1	17.0	63.6	162.7	23.9	27.7	5.6-5.3-1.3
Heptathlon	1	22.0	54.1	156.5	22.2	22.2	2.5-3.9-1.7
Indoor soccer	4	20.5	59.4	161.5	22.8	26.6	4.6-4.3-1.7
Indoor soccer, defender	4	20.8	56.5	161.5	21.8	23.9	4.1-4.0-2.3
Indoor soccer, forward	5	19.6	53.3	159.8	20.9	22.4	3.6-3.7-2.5
Indoor soccer, goalkeeper	4	20.0	64.4	158.8	25.6	30.2	5.9-4.9-0.8
Indoor soccer, midfielder	5	20.8	53.9	154.8	22.6	24.4	4.9-4.2-1.6
Javelin throw	1	22.0	76.2	163.5	28.7	29.2	4.9-5.7-0.2
Judo	6	19.8	55.4	161.2	21.3	21.9	3.2-4.3-2.4
Judo < 44 kg	1	20.0	44.1	145.0	21.0	23.6	5.2-4.5-1.5
Judo < 48 kg	1	20.0	48.7	152.0	21.1	23.5	4.5-4.6-1.9
Judo < 57 kg	2	20.5	56.9	160.5	22.1	23.0	3.2-4.1-2.0
Karate	8	18.5	58.8	158.9	23.1	26.9	5.0-4.4-1.6
Karate, kata	2	17.5	51.1	157.5	20.6	21.4	3.8-3.2-2.5
Karate, kumite	4	18.5	59.1	158.3	23.6	25.3	4.3-4.5-1.3
Kickboxing, low kick	3	19.3	57.8	163.0	21.8	25.7	4.6-3.4-2.3
Long jump	4	21.4	62.4	170.7	21.4	23.6	3.4-3.1-2.9
Olympic wrestling	18	19.1	58.2	157.1	23.5	24.6	4.6-4.9-1.4
Olympic wrestling < 53 kg	1	19.0	53.0	157.0	21.5	21.6	3.0-4.6-2.0
Padel	1	24.0	69.0	162.0	26.3	33.0	6.1-3.4-0.7
Padel, doubles, backhand player	1	21.0	59.2	154.0	25.0	27.7	4.3-5.2-0.7
Padel, doubles, right-handed player	1	23.0	57.2	164.0	21.3	25.5	3.5-3.4-2.6
Pole vault	1	23.0	60.9	164.0	22.6	23.6	2.6-4.8-1.9
Powerlifting < 44 kg	1	26.0	40.3	150.9	17.9	20.7	3.4-2.0-3.6

Powerlifting < 90 kg	1	17.0	88.6	167.0	31.8	37.8	7.5-6.6-0.1
Racewalking, 20 km	1	18.0	48.0	157.0	19.5	22.8	5.5-3.3-3.0
Road cycling	1	17.0	67.1	164.0	24.9	24.6	4.0-4.1-1.1
Rugby	6	19.5	59.4	158.5	23.6	26.3	4.8-4.2-1.4
Rugby 7s, prop	1	18.0	73.7	165.2	27.1	26.9	5.5-5.2-0.6
Rugby, center	2	19.0	52.6	156.5	22.3	24.9	4.8-4.0-1.6
Rugby, front row	1	27.0	74.6	160.8	29.1	33.6	4.6-6.4-0.1
Rugby, inside center	1	21.0	57.9	161.0	22.3	22.9	3.1-3.6-1.9
Rugby, prop	3	22.0	66.2	161.7	25.3	28.1	4.5-4.8-0.9
Rugby, scrum-half	2	19.5	54.0	156.8	23.0	25.8	4.6-4.2-1.6
Rugby, wing	7	20.6	52.9	158.6	20.6	22.9	3.6-3.6-2.6
Shot put	1	21.0	82.5	164.6	30.3	33.4	4.7-7.7-0.1
Soccer	30	20.7	56.4	159.3	22.3	23.9	3.9-4.3-1.9
Soccer, defender	20	19.2	55.2	161.3	21.2	22.4	3.8-3.8-2.5
Soccer, forward	13	20.5	60.5	161.7	22.6	24.5	4.1-4.1-2.0
Soccer, goalkeeper	7	19.6	65.0	162.5	24.6	27.9	5.4-4.5-1.2
Soccer, midfielder	10	20.1	54.0	155.7	20.9	22.1	3.5-4.1-2.3
Softball	11	19.8	60.3	161.8	22.9	26.3	4.5-4.0-1.8
Softball, fielder	3	18.0	59.5	164.0	22.1	24.2	4.6-3.7-2.2
Softball, second base	2	19.5	53.3	158.0	21.3	21.3	3.2-3.7-2.2
Softball, shortstop	1	20.0	60.5	163.0	22.8	26.1	4.1-4.5-1.8
Sport climbing	3	22.0	44.4	154.0	18.7	20.0	3.3-2.9-3.3
Sprint	7	19.2	54.9	162.4	20.8	20.5	2.8-3.6-2.9
Sprint, 100 m	1	21.0	57.7	162.0	22.0	22.7	3.4-3.4-2.1
Sprint, 200 m	3	23.0	60.5	164.2	22.5	23.6	3.8-3.6-2.0
Sprint, 400 m	2	23.0	51.9	162.0	19.7	20.9	3.0-2.8-3.2
Sprint, 400 m hurdles	1	18.4	63.8	163.7	23.7	21.9	2.6-4.6-1.4
Table tennis	7	20.4	60.5	160.8	23.6	26.7	5.1-4.5-1.9
Taekwondo	8	20.0	60.0	164.7	22.1	24.0	4.2-3.8-2.4
Taekwondo < 46 kg	1	22.0	48.4	162.0	18.4	18.1	2.4-2.0-4.0
Taekwondo < 49 kg	1	22.0	51.9	165.0	19.1	20.5	2.7-2.6-3.8

Track and field, 10,000 m	2	20.5	50.8	157.0	20.6	25.0	4.3-3.6-2.5
Track and field, 1,500 m	1	20.0	53.9	158.0	21.6	24.0	3.8-2.7-2.0
Track and field, 3,000 m steeplechase	1	20.0	47.1	155.0	19.6	18.5	3.1-4.9-2.8
Track and field, 4x100 m relay	1	20.0	59.0	162.0	22.5	22.8	3.1-3.9-1.9
Track and field, 5,000 m	1	22.0	49.9	156.4	20.5	20.6	2.6-4.8-2.5
Track and field, 800 m	3	19.0	53.4	157.1	21.6	21.2	3.0-4.1-2.0
Track and field long-distance	4	20.7	53.5	158.7	21.3	23.6	4.2-3.5-2.4
Track and field middle-distance	1	23.0	51.3	156.0	21.1	21.9	3.4-4.1-2.2
Track cycling	1	21.0	56.9	158.0	22.8	21.9	2.5-5.0-1.5
Triathlon	6	19.2	58.1	160.7	22.5	24.8	4.4-4.1-1.8
Volleyball	21	20.7	70.0	172.4	23.5	26.0	4.3-3.6-2.2
Volleyball, center	1	21.0	76.2	183.0	22.8	27.3	5.9-3.1-3.0
Volleyball, outside hitter	1	21.0	70.4	166.0	25.5	29.5	6.0-6.4-1.0
Volleyball, setter	1	19.0	66.1	167.0	23.7	24.5	3.3-4.9-1.7
Weightlifting	4	20.8	55.7	154.8	23.2	24.0	3.8-4.8-1.4
Weightlifting < 45 kg	1	22.0	47.6	155.0	19.8	18.7	2.1-4.5-2.7
Weightlifting < 55 kg	1	21.0	55.8	154.0	23.5	23.6	3.8-5.4-1.0
Weightlifting < 59 kg	2	18.5	62.8	152.0	27.2	28.5	5.2-6.8-0.2
Weightlifting < 64 kg	1	24.0	66.1	153.0	28.2	29.3	5.7-6.1-0.1
TOTAL	416						

Nota. The sample size (n), the mean values of the somatotype components (endomorphy, mesomorphy, and ectomorphy) according to the Heath-Carter method, body mass (kg), stature (cm), body mass index (BMI, kg/m²), and body fat percentage estimated using the equation proposed by Lean et al. (1996) are presented.
